# Genomic basis of fishing-associated selection varies with population density

**DOI:** 10.1073/pnas.2020833118

**Published:** 2021-12-13

**Authors:** Amélie Crespel, Kevin Schneider, Toby Miller, Anita Rácz, Arne Jacobs, Jan Lindström, Kathryn R. Elmer, Shaun S. Killen

**Affiliations:** ^a^Institute of Biodiversity, Animal Health and Comparative Medicine, University of Glasgow, Glasgow G12 8QQ, United Kingdom;; ^b^Department of Biology, University of Turku, 20500 Turku, Finland;; ^c^Department of Genetics, Eötvös Loránd University, Budapest H-1117, Hungary;; ^d^Department of Natural Resources, Cornell University, Ithaca, NY 14853

**Keywords:** fisheries, density-dependent effects, sequencing, anthropogenic effects, environmental change

## Abstract

Fisheries induce one of the strongest anthropogenic selective pressures on natural populations, but the genetic effects of fishing remain unclear. Crucially, we lack knowledge of how capture-associated selection and its interaction with reductions in population density caused by fishing can potentially shift which genes are under selection. Using experimental fish reared at two densities and repeatedly harvested by simulated trawling, we show consistent phenotypic selection on growth, metabolism, and social behavior regardless of density. However, the specific genes under selection—mainly related to brain function and neurogenesis—varied with the population density. This interaction between direct fishing selection and density could fundamentally alter the genomic responses to harvest. The evolutionary consequences of fishing are therefore likely context dependent, possibly varying as exploited populations decline. These results highlight the need to consider environmental factors when predicting effects of human-induced selection and evolution.

## Significance

Fisheries-associated selection is recognized as one of the strongest potential human drivers of contemporary evolution in natural populations. The results of this study show that while simulated commercial fishing techniques consistently remove fish with traits associated with growth, metabolism, and social behavior, the specific genes under fishing selection differ depending on the density of the targeted population. This finding suggests that different fish populations of varying sizes will respond differently to fishing selection at the genetic level. Furthermore, as a population is fished over time, the genes under selection may change as the population diminishes. This could have repercussions on population resilience. This study highlights the importance of selection but also environmental and density effects on harvested fish populations.

The selective harvest of animals by humans is one of the most important contemporary pressures on natural populations (1, 2). Intensive commercial fishing has been demonstrated to alter life history traits (e.g., reduced body size and/or age and size at maturation) in ecologically and economically important populations (3–7). However, a major question persists about whether the observed changes stem from Darwinian evolution via selection on phenotypic traits and associated genotypes or result from human-induced environmental changes generating phenotypic plasticity (8–11). In addition, harvest-associated phenotypic plasticity may interact with the fishing selection on genotypes (Gene by Environment interaction, G×E) to alter evolutionary outcomes, but this possibility has been overlooked.

For selection by fishing to occur, there must be phenotypic variation among individuals with respect to their vulnerability to capture. Vulnerability is likely comprised of a suite of life history, morphological, physiological, and behavioral traits that interact to determine whether a fish will ultimately escape or be captured by a fishing gear. While size and maturation are well established to be selected by fishing (12, 13), emerging evidence shows that fishing may also drive selection on traits related to bioenergetics or social behavior that also vary widely within species (14–16). This is especially likely given that commercial fishing methods such as trawling directly exploit aspects of fish foraging, schooling, and escape behaviors to facilitate capture (14). If the traits under fishing selection possess a genetic basis, fishing could lead to direct evolution (8, 17). Recent research suggests that fisheries can induce a shift in the genomic variants of targeted populations (18–20). While an analysis of wild populations would require more detailed time series and genomic data to securely infer the genomic responses to fishing (11), previous experimental work has mainly examined responses to size-based selection with no attempt to determine whether vulnerability to capture as an integrated trait can indeed select on specific genotypes and underlying genomic variants. Such genomic information is potentially valuable for predicting the consequences of selective harvest on targeted populations with the benefit of understanding which molecular changes might be involved and fuel evolution.

Harvest-associated plasticity could also occur in response to environmental effects because fishing causes other confounding environmental changes that could influence phenotypic expression (9, 21), such as the reduction of population density over time. Indeed, intense harvesting can remove so much biomass from the environment that the density for the remaining population is altered. A reduced population density may then decrease interindividual competition and correspondingly increase resource availability or alter among-individual variation in resource acquisition (22). Such conditions may not only modify the average phenotype of the remaining population and reduce phenotypic variation within the population (23) because of more homogenous food allocation among individuals, but also affect which individuals have a selective advantage in that new context. Different phenotypes and genotypes may therefore be selected by fishing pressures depending on population density, creating G×E effects to produce new selective landscapes and evolutionary trajectories for the remaining population. Previous modeling studies (24, 25) highlighted the importance of considering the contribution of population density in harvest-induced evolution, but, so far, empirical studies examining how population density reduction can affect selection and evolutionary potential in a fisheries context are lacking. Increased knowledge of the independent and interactive roles of direct selection by fishing and density-dependent effects is critical for understanding the integrated possible evolutionary consequences of fisheries on natural populations and for devising well-informed and sustainable strategies for harvest management.

## Results

### Experimental Selection on Phenotypes.

To address these issues, which are intractable in wild populations, we used an experimental approach using scaled-down gears and a surrogate species under varying population densities. The use of surrogate species has already been advocated by several authors (17, 26). Zebrafish (*Danio rerio*) have similar behavior (such as exploration, sociability, and shoaling) as larger fish species targeted by fisheries, reproduce easily in captivity, and thus are a suitable surrogate species for experimental studies of fisheries-induced evolution (26). We created 36 families of semiwild zebrafish, with each family equally split at hatching into either a population of baseline density [i.e., the density recommended for zebrafish rearing (27)] or a population of reduced density (half that of the baseline). After 6 mo, 10 fish per family per density were used to create our experimental populations (360 fish per density), with the baseline density fish being housed in two 55-L tanks and the reduced density fish being housed within four 55-L tanks. Each tank was further subdivided into four sections using transparent dividers. Each fish was then screened for a range of life history, physiological, and behavioral phenotypic traits, including size, growth rate, aerobic metabolic rate, aggression, and sociability. The fish were then submitted to scaled-down trawling simulations repeatedly over six fishing trials that comprised a total of 75 individual fishing events to mimic commercial fisheries that gradually harvest fish over time (*SI Appendix*, Fig. S1). A key advance of our study is that we quantify vulnerability to capture as an integrated trait as opposed to previous work that has mainly selected only on body size during experimental harvest (18, 19). The 20% most vulnerable fish (based on the shortest time to be caught in the first trawling event, i.e., captured fish) and 20% least vulnerable fish (escaping the last trawling event and were never captured over the course of the trawling events, i.e., escaped fish) were identified at the end of trawling simulations (*n* = 72 fish per vulnerability and population density).

**Fig. 1.**
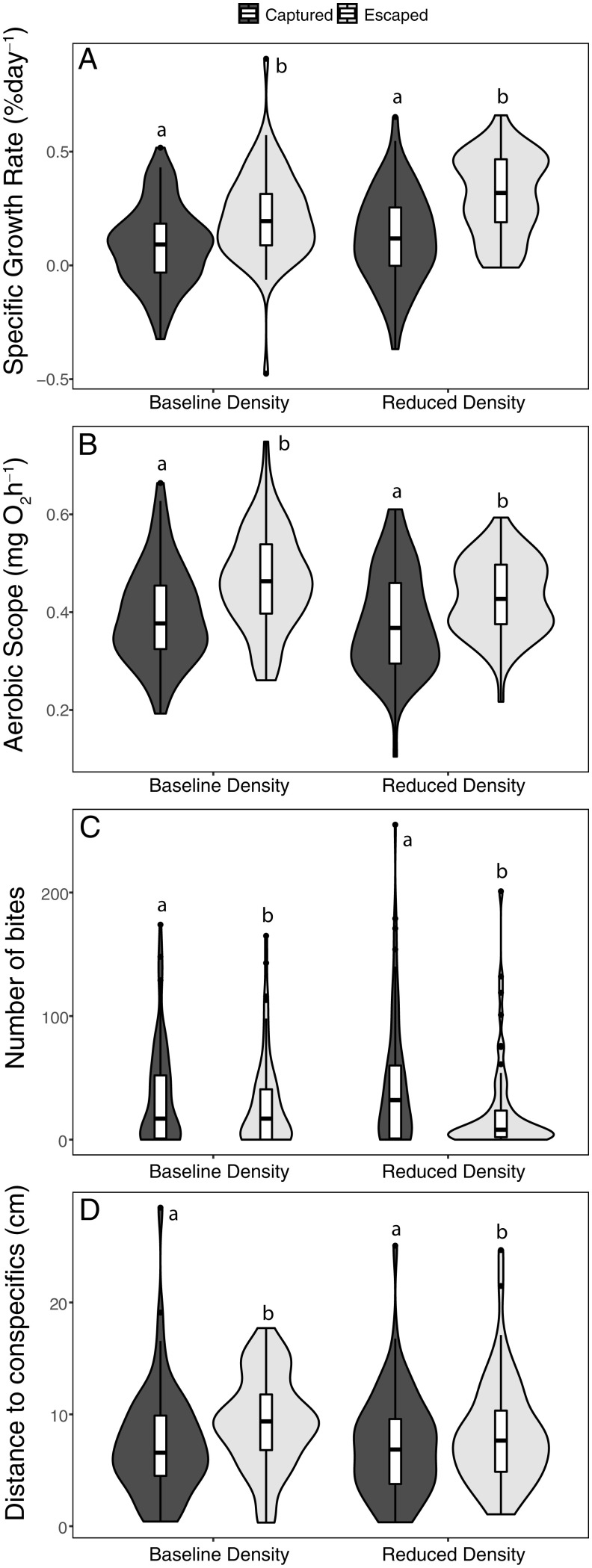
Physiological and behavioral phenotypic selection in the two density populations. The distribution of the specific growth rate (*A*), aerobic scope (adjusted to the mean mass of the fish, i.e., 0.30 g) (*B*), level of aggressiveness (*C*), and sociability (*D*) of captured (dark gray) and escaped fish (light gray) after a series of trawling simulations reared either under a baseline or reduced density (*n* = 75 per group). Different letters indicate significant difference among the conditions (GLM: *P* < 0.05).

We observed that the trawling simulation selected for similar phenotypic differences between the captured and escaped fish regardless of population density (Fig. 1). A general linear model (GLM) multivariate analysis including all life history, physiological, and behavioral traits measured revealed a significant difference between the captured and escaped fish (multivariate GLM: F4,238 = 9.09, *P* < 0.0001). The phenotypic differences between captured and escaped fish were similar across the population densities; no interaction between vulnerability and density (multivariate GLM: F4,238 = 0.91, *P* = 0.46) and no main effects of density were observed (multivariate GLM: F4,238 = 1.60, *P* = 0.17). Further analysis of the differences using individual traits revealed that the escaped fish had a higher specific growth rate (∼150% faster growth, GLM: F1,285 = 10.31, *P* = 0.001, Fig. 1*A*), higher aerobic metabolic scope (∼16% higher, GLM: F1,286 = 7.55, *P* = 0.006, Fig. 1*B*), lower aggression (∼37% fewer bites toward mirror reflection, GLM: F1,262 = 4.57, *P* = 0.034, Fig. 1*C*), and lower sociability (∼21% further from conspecifics in sociability assay, GLM: F1,263 = 3.94, *P* = 0.048, Fig. 1*D*) than the captured fish, regardless of density.

### Experimental Selection on Genome.

To determine the potential evolutionary effects of this fisheries-induced selection on phenotype and investigate the broad range of traits that could be under selection, we screened for differential selection on genetic variants using low-coverage (∼2× coverage per individual), whole-genome sequencing. We sequenced 24 fish (siblings from the same family origin across the groups) from high and low vulnerability to capture in the baseline and reduced density populations (*n* = 4 × 24 = 96 in total). We examined the differential allele frequency of the genomic variants using genotype likelihoods (28).

**Fig. 2.**
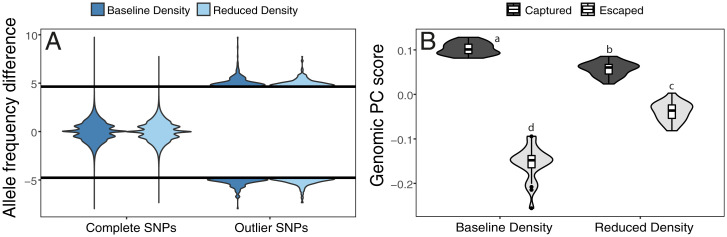
Genomic variation and selection in the two density populations. (*A*) Distribution of the allele frequency difference between captured and escaped fish after a series of trawl simulations, with the complete set of SNPs (5 666 304 SNPs) and the outlier SNPs (480 and 517 SNPs in the baseline and reduced density respectively) in the fish reared under baseline (dark blue) or reduced (light blue) density. The dark line represents the outlier threshold based on the 0.5% quantiles of the Bonferroni-corrected zdAF (4.65 and −4.77, upper and lower cutoff, respectively). (*B*) Distribution of the genomic PC score of the outliers of captured (dark gray) and escaped fish (light gray) after a series of trawling simulations, reared either under a baseline or reduced density (*n* = 24 per group). Different letters indicate significant difference among the conditions (GLM: *P* < 0.05).

**Fig. 3.**
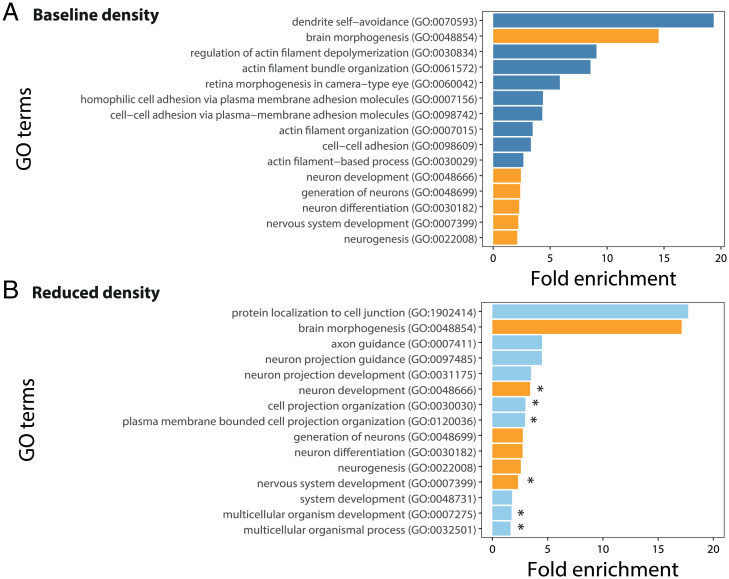
Outlier GO terms from the genes selected by the fishing simulation in the two density populations. The significance and fold enrichment of the 15 most significant GO terms are represented in the outliers of the fish reared under a baseline (*A*) or reduced (*B*) density. The numbers shown in parentheses are the GO identities of each biological process and GO term. Dark blue GO terms represent GO terms only present in the baseline density population, while clear blue GO terms represent GO terms only present in the reduced density population. Orange GO terms represent the GO terms present in the two populations. GO terms with asterisks are the GO terms with significant enrichment (FDR < 0.05). The complete list of the outlier GO terms from each population is available in *SI Appendix*, Table S1.

Our analysis of over 5.67 million reference genome–mapped single-nucleotide polymorphisms (SNPs) indicated that the trawling simulation had a selective effect at the genomic level that was consistent across families within each density. We observed an expected heterozygosity (H_e_) of 0.25 for all samples combined, which is in the range of natural zebrafish populations (29). Using a restrictive threshold based on random permutation (upper and lower global 0.5% quantiles [z-transformed differences in allele frequencies, i.e., zdAF, = 4.65 and −4.77, respectively] of 0.05% Bonferroni-corrected z-transformed allele frequency differences from each SNP), we detected the outlier SNPs with allele frequencies that significantly differed between the captured and escaped fish across the families in each population density (Fig. 2*A*). We identified 239 annotated SNPs in 220 genes or in noncoding regions and 241 unannotated SNPs that significantly differed between the captured and escaped fish in the baseline density. In the reduced density, 268 annotated SNPs in 239 genes or in noncoding regions and 249 unannotated SNPs were significantly different between captured and escaped fish. By targeting several hundred genes, this fisheries-induced selection follows the classic quantitative genetic prediction of selection on complex traits (30). The outlier genes identified were mainly involved in brain function and neurogenesis (Gene Ontology [GO]; Fig. 3 and *SI Appendix*, Table S1). Similarly, the gene set enrichment analysis based on the z-transformed allele frequency differences of all SNPs (not only the outliers) also indicated trends of enrichment for nervous system processes (*SI Appendix*, Fig. S2). The trawling simulation seems therefore to induce additional selection on the neurological functions of the fish.

**Fig. 4.**
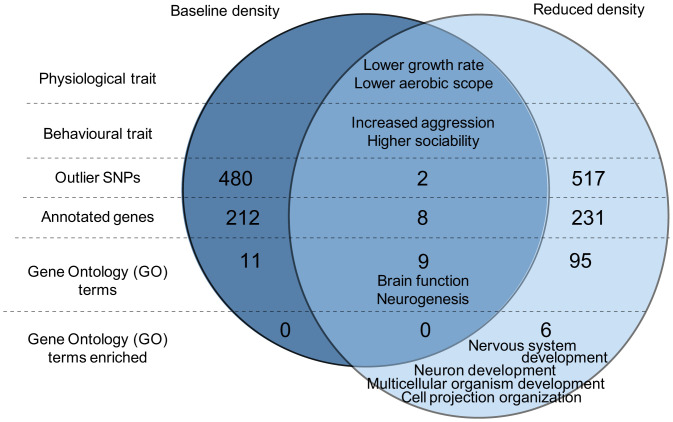
Summary of the phenotypic and genomic selection by the fishing simulation according to population density. Shown are differences in captured fish relative to those that were never captured, illustrating the selection by fishing on the phenotypes (physiological and behavioral traits) and the genomes (outlier SNPs, annotated genes, GO terms, and enriched GO terms) of fish reared at a baseline (dark blue) or reduced (light blue) density. The overlapping section represents the selection that is shared between the baseline and reduced density. The complete list of the GO terms from each population is available in *SI Appendix*, Table S1.

Notably, however, the genes and particular biological functions potentially selected by the trawling simulation differed depending on population density (Fig. 3 and *SI Appendix*, Table S1). From the outlier SNPs identified as different between the captured and escaped fish in each population density (480 in the baseline density and 517 in the reduced density), only two overlapped between the baseline and reduced density populations (one annotated and one unannotated, *SI Appendix*, Table S2), which is significantly fewer than expected by chance (Fisher’s exact test, *P* < 0.0001). From the genes identified with the outlier SNPs of each population density, only eight (mainly involved in brain and eye development) were shared between the baseline and reduced density (*SI Appendix*, Table S2), which is similar to that expected by chance (Fisher’s exact test, *P* = 0.10), and only one of those genes involved an overlapping SNP. Further confirmation of the density effects on genomic response, based on replication and reanalysis within experimental groups, also found that between-density differences were greater than within-density differences (*SI Appendix*, Tables S3 and S4) both in number of outlier genes and in gene functions. The proportion of the different genomic variants (e.g., missense variant, synonymous variant, etc.) represented in the outliers of each population density were similar across density (*SI Appendix*, Table S5). A mere nine GO terms overlapped between the baseline and reduced density populations, with only six in the 15 most significant GO terms (Fig. 3 and *SI Appendix*, Table S1). In addition, only six GO terms in the reduced density were significantly enriched in alleles different between the captured and escaped fish (false discovery rate [FDR] corrected: nervous system development, neuron development, multicellular organismal process, multicellular organism development, plasma membrane bounded cell projection organization, and cell projection organization), while none were significantly enriched in the baseline density (Fig. 4). These results are a strong indication of an interaction between the population density and the allele selection induced by the trawling simulation.

Genomic multivariate analysis revealed strong differential selection by trawling at the genome level, which differed between population densities. Using the allele frequency difference of all outlier SNPs from the two population densities, we ran a multivariate analysis (principal component analysis [PCA]) separating escaped from captured individuals (31% of variance explained) and extracted a genomic principal component (PC) score for each fish. The genomic PC score was then used to represent the overall genome of each individual. A significant interaction between vulnerability and density was observed on the genomic PC score obtained (GLM: F1,88 = 241.8, *P* < 0.0001). The difference in the genomic PC score between captured and escaped fish was more extreme at baseline density, while escaped fish had a lower genomic PC score in both density conditions (Fig. 2*B*). In addition, the genomic PC score of the escaped fish in the baseline density was significantly lower than the genomic PC score of the escaped fish from the reduced density (Fig. 2*B*). An additional analysis conducted replicating within density (*SI Appendix*) also revealed that no difference was observed between groups of the same density (*SI Appendix*, Figs. S3 and S4). These results again point to the conclusion that trawling-induced selection at the genomic level differs depending on the prevailing population density. The analysis also highlighted a stronger similarity within each density/vulnerability group than across. The same families were represented in each group, suggesting stronger effects of vulnerability and density compared to family on the presence of outlier genomic variants, meaning that the trawling selection on the genomic variants was also consistent across the different families.

### Genotype–Phenotype Association.

**Fig. 5.**
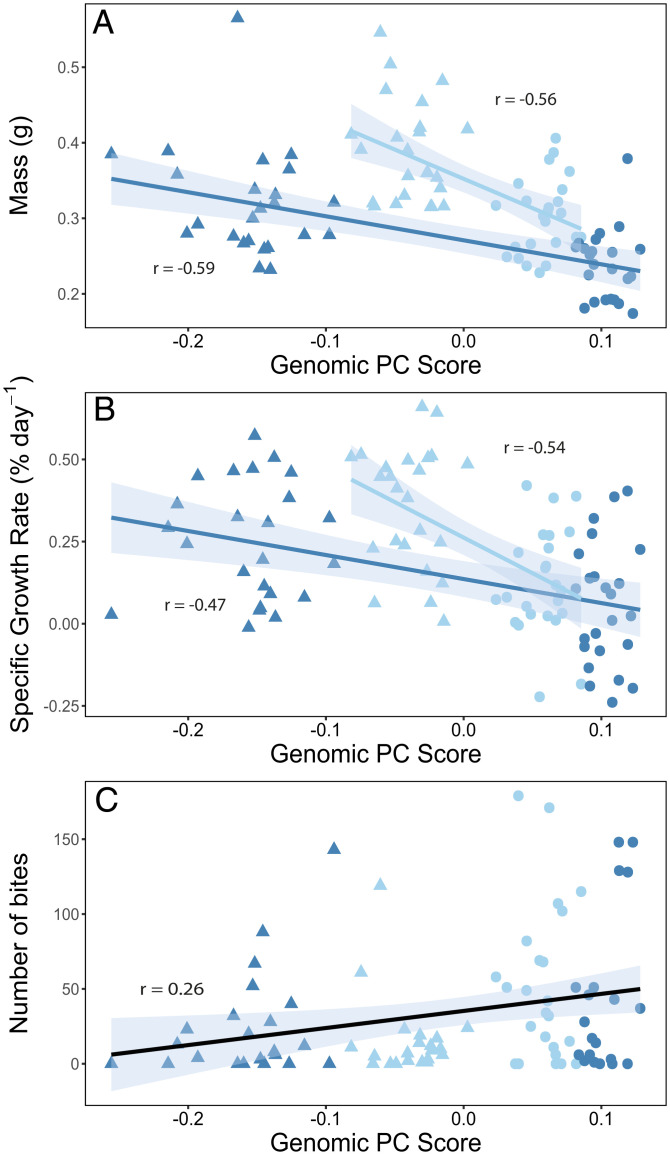
Significant correlations between genomic and phenotypic variance. The correlations between the genomic PC score of the outliers and mass (*A*), specific growth rate (*B*), and aggressiveness (*C*) of captured (round shape) and escaped (triangle shape) fish after a series of small-scale trawling simulations reared either under a baseline (dark blue) or reduced (light blue) density (*n* = 24 per group). The black line in *C* represents the main effect of genomic PC score (there was no interaction with density). The shaded areas around the lines correspond to 95% intervals.

The genomic PC score correlated with some of the measured phenotypic traits (Table 1 and Fig. 5), revealing genotype–phenotype associations. Significant correlations were observed between the genomic PC score and body mass (Pearson’s correlation: baseline density *r* = −0.59, *n* = 48; reduced density *r* = −0.56, *n* = 48; *P* < 0.001 for both), specific growth rate (Pearson’s correlation: baseline density *r* = −0.47, *n* = 48; reduced density *r* = −0.54, *n* = 48; *P* < 0.001 for both), and aggression (Pearson’s correlation: combined densities *r* = 0.26, *n* = 96, *P* = 0.015). Even though a significant density effect was observed on the correlation between the genomic PC score and the mass or specific growth rate (Table 1), the direction and strength of the correlation was similar between densities. These correlations suggest that these phenotypic traits, which are under fishing selection, possess a genomic basis also under selection by the fishing process. Evolution in response to harvesting could thus be expected for these traits.

**Table 1.** Correlations between genomic and phenotypic variance

Correlation with genomic PC score	Combined densities			Interaction with density	Baseline density			Reduced density		
	*r*	*r*²	*P* values		*r*	*r*²	*P* values	*r*	*r*²	*P* values
Mass				**0.012**	**−0.59**	**0.35**	**<0.001**	**−0.56**	**0.31**	**<0.001**
SGR				**0.009**	**−0.47**	**0.22**	**<0.001**	**−0.54**	**0.29**	**<0.001**
Aerobic scope	−0.09	0.01	0.372	0.495						
Aggressiveness	**0.26**	**0.07**	**0.015**	0.167						
Sociability	−0.19	0.04	0.069	0.826						

*r* is the Pearson coefficient of correlation, *r*² the coefficient of determination, phenotypes in bold are significantly correlated with the genomic PC score (Pearson’s correlation: *P* < 0.05). *n* = 96 in the combined densities, *n* = 48 in the baseline and reduced density.

## Discussion

Our results show the potential for an important, but to date overlooked, interaction between harvest-associated selection and environmental harvest-associated effects, measured here as a reduction in population density, on the selection of genotypes. Specifically, even though selection at the phenotypic level imposed by trawling was similar in the two density conditions, selection at the genomic level acted on different underlying genes depending on the population density (Fig. 4). Harvest-associated reduction in population density could thus fundamentally shift the evolutionary trajectory of targeted populations at the genomic level. It is therefore imperative to consider that the harvest of wild individuals has the potential to not only alter the phenotypes and genotypes present within a population through direct selection but also through associated density-dependent effects on the underlying genes under selection. This combination of effects could further limit our ability to predict the evolutionary consequences of fisheries, especially as the density of targeted populations will tend to decrease through time, potentially regularly shifting the selection on genomic variants present even if selection on phenotypes remains constant.

Fish with a lower specific growth rate were more vulnerable to our simulated capture. The predicted responses of fishing selection on growth rate are complex and depend on a number of factors, including any thresholds for size-based selection (e.g., length limits at which fish can be retained because of management-based size restrictions) and energy investment before versus after maturity (3, 5, 19, 24). Importantly, our study contained no a priori assumption of size-based selectivity and instead considered vulnerability to capture as an integrative trait unto itself. Therefore, faster growing fish may have been better able to escape the trawl because of increased swimming endurance or higher absolute swimming speeds. This suggests that trawling may produce selection on growth rates, perhaps because of correlations among growth rate and components of locomotor ability and bioenergetics, which can be separate from the selective pressures on growth stemming from size-selective mortality.

Our experimental trawling simulation selected not only on life history traits (specific growth rate) but also on physiological (aerobic metabolic rate) and behavioral (aggression and sociability) traits similarly in both density conditions. This finding provides further evidence that important physiological and behavioral traits in addition to body size can be directly targeted by fishing and could determine the capacity of an individual to be captured by a particular fishing gear or technique (14, 16, 31, 32). For example, higher aerobic metabolic scope probably allows a fish to reach a faster swimming speed or have greater swimming endurance, enabling it to out swim a trawl (14). Similarly, more social fish could be more likely to follow conspecifics into the net when groupmates tire and get captured rather than leaving the group to find a way to escape (14). Selection on particular phenotypes could thus lead to a shift in the phenotypic composition of the remaining population. Especially as a number of these phenotypic traits seem also to possess some heritability, this could lead to differential evolution for the targeted population (8).

From a genomic perspective, our trawling simulations affected hundreds of genes, mainly associated with brain functioning and neurogenesis, in both density conditions. Any traits or other important biological functions associated with these genes could therefore also be targeted by the fisheries process in a manner that not only depends on selection by the fishing gear but also on the density of the harvested population. Our SNP analyses thus highlight that further attention should be given to the involvement of brain structure or individual cognition in the capacity of fish to escape fishing gears. Nonetheless, fishing has the potential to induce a genomic change by selection on specific SNPs, which can ultimately lead to the evolution of the population. However, our experiments do not allow conclusions about how the trajectory of selection might change in subsequent generations.

Even though the fishing simulations selected similar functions and phenotypic traits in both density conditions, specific genes and genomic regions were differentially selected in the different conditions. The possible evolutionary consequences of fishing may thus not be predicted from phenotypic observations alone (18), as changes in some phenotypic traits, such as those associated with neurological functions, and their ecological and evolutionary implications may be difficult to assess. The low genomic repeatability between the populations of different density and the subtle allele frequency shifts of many loci also highlight the high genetic redundancy and polygenic basis of the escaped phenotype (33), suggesting many different combinations of genetic changes can lead to a higher chance of escaping (34, 35). These results are concordant with the analysis of Pinsky et al. (11) that did not find strong signals of fishing-selective genomic trace in overfished cod populations. They explained that these results were either because of density-dependent phenotypic plasticity or polygenic selection with subtle allele frequency changes, both of which we show as indeed being of major importance for fishing-induced selection. Population density may affect how fish experience intraspecific competition and other among-individual interactions (22), potentially affecting the level and quality of external sensory stimuli received by each individual and, therefore, their brain development (36). Therefore, depending on the density of a targeted population, distinct genomic pathways may be under selection by fishing, potentially leading to divergent evolutionary trajectories over time. As intense harvesting may be accompanied by a pronounced reduction of population density, the interaction between harvest-associated genomic selection and density-dependent environmental effects (G×E) is likely to occur in targeted populations and potentially shift the evolutionary outcome of the fishing process. The presence of G×E could thus limit the strength of the fishing selection through time, selecting different genomic regions when population density is reduced, potentially maintaining genetic diversity as previously reported (11). Alternatively, such G×E could also threaten the resilience of populations to further harvesting pressure or environmental challenges because of the selection of potentially maladaptive genomic variants or unexpected correlated changes on other phenotypic traits initially believed to be unrelated to fishing. The greater the density reduction in the targeted population, the greater the probability of density-dependent G×E interactions. At low densities, populations are also at increased risk of experiencing Allee effects, which occur when the per capita population growth rate (and average fitness of individuals within the population) declines as abundance decreases (37, 38). Such Allee effects could additionally limit the rate of recovery of the targeted populations and have nonlinear consequences that are challenging to predict (25, 37).

The genes under selection that were involved in brain function and cognition might underlie the differences observed in growth and aggression between captured and escaped fish. For example, an enhancement of cognition could lead to improved food finding, foraging, and competitive ability (5, 39), which in turn could lead to faster growth. The absence of correlations between genomic PC score and either aerobic metabolic scope or sociability, despite these phenotypic traits being under selection in the trawling simulations, suggests that these traits may not possess a clear genomic basis that would be targeted by fishing selection or, alternatively, are highly polygenic with fitness effects spread across a large number of genomic variants. Other factors (probably more environmental than genotypic, such as the presence of conspecifics in the net or training effects on aerobic capacity) could have also influenced the contribution of these traits to fish vulnerability to capture and may also play a role in fishing in general.

It is important to consider the similarities and differences between our experimental setup and actual trawl selection. Swim flumes have previously been used as a tool to study fish vulnerability to trawling (40, 41) and mimic the tendency of fish to hold station at the mouth of an approaching trawl net (42). A notable difference is that real trawls can target hundreds or thousands of fish simultaneously, while we were limited to the number of fish we could test within a given trial. Increased numbers of individuals could enhance the importance of social interactions for vulnerability to capture. Fish in real trawls may have additional opportunities for escape either above or around the net or beneath the ground gear. While we simulated these escape routes, it is possible that differences in escape mechanics may alter the traits under selection in addition to traits associated with swimming performance. It is notable, however, that fish in our trawl simulation used escape routes in a manner similar to that observed in real trawls (43). Finally, the current study focused on the critical final stage when the fish have encountered the gear and attempt to escape. Actual harvest-induced selection may integrate various additional steps which will determine an individual's overall capture vulnerability (14), including habitat use by individual fish and gear encounter rate (14, 44). Additional work is required to understand how the various stages of the capture process may further affect which traits and genes are under selection.

## Conclusion

The present findings suggest a one-fits-all evolutionary approach would not be appropriate for the management of wild populations that are subjected to harvest by humans (7). Instead, a more integrative approach that considers both direct human-induced selection and other environmental effects such as population density is necessary. It is critical that both genomic and ecological factors must be considered to fully understand and predict the consequences of human-induced selection on the resilience of natural populations. This is because the outcome of selection in one environment will most likely not be representative of the outcome of selection in another environmental context (45). Our results have also wide implications for studying the interplay of genetic and ecological factors in determining possible evolutionary outcomes in a broader ecological context, for example, in case of predation, or the interaction between sexual selection and population fluctuations.

## Materials and Methods

### Experimental Population.

In 2017, a semiwild zebrafish (*Danio rerio*) population sourced from rearing ponds in Malaysia (JMC Aquatics) was transferred to the University of Glasgow. A total of 24 adults were used to produce 36 families in a controlled factorial (North Carolina II) breeding design, where four groups of three males were reciprocally crossed to three females. After hatching (at 4 d postfertilization), each family was separated equally into two densities: a baseline density (60 larvae/L) and a reduced density (30 larvae/L). The families were then transferred and kept separated in 2-L tanks held under a 13-h light : 11-h darkness photoperiod and supplied with recirculating dechlorinated filtered freshwater maintained at 28 °C. The larvae were fed four times daily with a combination of commercial food (TetraMin baby, ZM fry food, Zebrafeed, Novo Tom) and live *Artemia nauplii*. After 2 mo, we estimated about 10% mortality within families for both density conditions and readjusted the density number of fish (baseline density 40 juveniles/L and reduced density 20 juveniles/L). When the fish reached 6 mo, 10 fish per family and density (i.e., 360 fish per density) were randomly chosen within the tanks and tagged using a visual implant elastomer (Northwest Marine Technology) with a unique code identifier of four colors on the dorsal region (46). The families were then mixed and transferred into 55-L tanks divided into four equally sized sections (each being around 13.5 L) supplied with recirculating dechlorinated filtered freshwater maintained at 28 °C (*SI Appendix*, Fig. S1). The density conditions were then adjusted to 6 fish/L [baseline density (27)] and 3 fish/L (reduced density). The fish were fed twice daily with a combination of commercial food (TetraMin Tropical Flakes, ZM small granula) and live *Artemia nauplii*. Less than 1% mortality was observed within families and tanks during this period of rearing for both densities. The rearing conditions thus unlikely represented an initial source of genomic selection. Before every manipulation, the fish were fasted for 24 h.

### Phenotypic Characterization.

#### Growth.

During the tagging (6 mo old) and at 9 mo old, the fish (*n* = 360 per density) were measured for their body mass (to the nearest milligram) and fork length (to the nearest 0.01 mm), and their sex was determined. The specific growth rate (SGR) of each fish was calculated according to the formula SGR = Ln (m_f_ – m_i_)/T, in which m_f_ is the mass (g) of the fish at 9 mo, m_i_ is the mass (g) of the fish at 6 mo, and T is the time in days between the two measurements.

#### Respirometry.

Individual fish oxygen uptake (MO_2_) was measured using intermittent flow respirometry as previously described (47, 48). Briefly, the setup was immersed into a 40-L tank filled with fully aerated freshwater thermoregulated at 28 °C and shielded from surrounding disturbances. The setup comprised 16 glass chambers (22 mL) connected to oxygen probe holders in a closed recirculating loop using a peristaltic pump. The closed circuit insured good mixing of the water and allowed the monitoring of the oxygen level in the chambers using FirestingO2 optical oxygen meters and probe sensors (PyroScience GmbH) calibrated daily inserted in the probe holders. Submersible pumps (Eheim GmbH) supplied fresh fully aerated water into the chambers for 2 min every 10 min creating measuring cycles.

Individual fish were placed in a 30-L swimming tunnel (Loligo Systems) and forced to swim until exhaustion (i.e., when no longer able to swim against the flow) for 2 min. The fish were then rapidly placed into a respirometry chamber to measure their maximum metabolic rate (MMR) postexercise. The fish were maintained in the chambers overnight (i.e., about 15 h) to estimate their standard metabolic rate (SMR). The fish were then removed from their chambers, measured for their mass and length, and returned to their rearing tanks. Blank oxygen consumption was measured in the empty chambers before and after the measurements of the fish to estimate bacterial respiration.

Fish MO_2_ (mg ⋅ O_2_ ⋅ h^−1^) was calculated using the slopes of decline in oxygen in the chambers measured in LabChart multiplied by the volume of the chambers minus the volume of the fish and corrected by the background bacterial respiration. Fish SMR was determined as the 0.2 quantile of the MO_2_ measurements (49). The fish MMR was determined as the maximum MO_2_ obtained during the 30 min after the swimming exercise. The aerobic scope (AS) was determined as the difference between MMR and SMR.

#### Aggressiveness.

The aggressiveness of each fish was measured using a mirror assay. The setup comprised 16 individual square tanks (17 × 17 cm) filled to a 5-cm depth with aerated freshwater thermoregulated at 28 °C and shielded from surrounding disturbances. The fish were acclimated in the empty tanks for 10 min. The mirror (8.5 × 30 cm) was then introduced on one side of the tank, and the fish behavior was recorded for 10 min using four webcams (Logitech HD Pro C920) and iSpy software (iSpyConnect). The videos were then analyzed using Ethovision XT 11 (Noldus, 2001), and the total number of bites against the mirror was quantified and used as a proxy for the fish’s aggressiveness.

#### Sociability.

The sociability of each fish was measured using four rectangular glass tanks subdivided in three sections with a central focal section (32 × 19 cm) and two side sections (13 × 19 cm) separated by transparent acrylic. The tanks were filled to a 10-cm depth with aerated 28 °C freshwater and shielded from surrounding disturbance. Between each trial, 50% of the water was changed to maintain the water temperature and oxygenation level. At the beginning of the trial, a group of stimulus fish (three males and three females unfamiliar to the focal fish) were placed randomly in one of the side sections and left to acclimate for 5 min. The other side section remained empty. The focal fish was then placed in the central focal section within a transparent cylinder placed in the middle of the section to acclimate for 5 min. The cylinder was then removed, and the fish behavior was recorded for 20 min using two webcams (Logitech HD Pro C920) and iSpy software (iSpyConnect). At the end of the trial, the fish were removed, their mass and length measured, and returned to their holding tanks. The videos were then analyzed using Ethovision XT 11 (Noldus, 2001), and the average distance of the focal fish to the stimulus group of fish was determined and used as a proxy for fish sociability.

### Fisheries Simulation.

The trawling simulations took place after the phenotypic characterization in a 90-L swimming tunnel (Loligo Systems) at 28 °C shielded from surrounding disturbance. A 30-cm-long small-scale custom-designed model trawl net (designed by the Fisheries and Marine Institute of Memorial University of Newfoundland) with escape routes on the upper side areas of the net mouth was used for the simulations (*SI Appendix*, Fig. S1). For each trawling event, fish from either the baseline or reduced density were acclimated in groups of 16 in front of the trawl hidden by a separator at a water velocity of 4 cm ⋅ s^−1^ for 20 min. After the acclimation, the separator was removed, and the water velocity was rapidly increased (over 30 s) to 50 cm ⋅ s^−1^, the lowest velocity at which individuals shift to anaerobic swimming (i.e., upper limit of sustainable swimming as is the case in an actual trawling event) (50). The event lasted for 10 min, during which the time the fishes captured by falling back in the trawl were recorded to determine vulnerability to trawling capture. Fish that reached the end of the net passed through a tube and into an acrylic compartment where they were shielded from the oncoming flow. This simulated being captured in the codend but allowed the fish to be retained without being compressed against the net. At the end of the event, the position of the fish (captured in the net or acrylic compartment, or that escaped either in front or behind the net) was also recorded. Once the entire population had passed through the first trial (*n* = 360 fish per density, 22 events in the first fishing trial), the 20% of fish that were most vulnerable in each density (*n* = 72 per density) were identified according to their time of capture and were removed from the experimental populations. The experimental fish were then returned to their rearing tank randomly. The trawling simulations with the new populations were repeated every week for 6 wk in total (six fishing trials consisting of 75 fishing events in total), identifying and removing each time the 20% of fish that were most vulnerable to capture. At the end of the 6-wk period, the 20% of fish least vulnerable to capture in each density (*n* = 72 per density) were those that had escaped every trawling simulation. These “escaped fish” together with the 20% “most vulnerable” fish to trawling capture (i.e., those captured in the first trawling simulation) were then considered to be our vulnerability groups.

### Genomic Analyses.

#### Sample collection, DNA extraction, library preparation, and sequencing.

After the fisheries simulation, a fin clip was taken from 24 individuals from each vulnerability group under each density (total *n* = 96), with the 24 individuals balanced from the families present in all the groups (*SI Appendix*, Table S6). The MagMax DNA Multi Sample Kit (Applied Biosystems) was used to extract high molecular weight DNA from tissue. The concentration, purity, and integrity of the DNA extractions were assessed using the Qubit double-stranded DNA broad range (dsDNA BR) assay (Thermo Fisher), the Nanodrop (Thermo Scientific), and electrophoresis on 1% agarose gel. A barcoded library for each individual was prepared using NEBNext Ultra II FS DNA Library Prep Kit for Illumina (BioLabs.) reagents and protocols. Briefly, 100 ng input DNA samples were digested over 20 min followed by adapter ligation. The products were then cleaned and size selected (250 to 500 base pair [bp]) using Agencourt AMPure XP beads. PCR was used to add the unique dual index barcodes and amplify the libraries over seven cycles. The libraries were then combined equally and purified using Agencourt AMPure XP beads. The final concentration of the library was quantified (9 ng/µL) using the Qubit dsDNA BR assay (Thermo Fisher), and the fragment size distribution was assessed using high-sensitivity DNA assay on an Agilent Bioanalyzer instrument (average size 325 bp). The library was sequenced on four lanes of Illumina HiSeq X Ten (BGI) with paired-end 150-bp reads.

#### Data filtering, mapping, and genotype likelihood calculation.

The raw reads were filtered to remove potential lower quality reads and artifacts using Trimmomatic v0.36 (51) and cutadapt v1.16 (52). The reads were aligned and mapped to the zebrafish reference genome (GRCz11) using the mem algorithm of Burrows–Wheeler Aligner software (BWA v0.7.17) (53). Sequence duplicates were removed with MarkDuplicates in Picard v2.18.14 (54). The coverage per individual was 2× ± 0.5 in the final dataset. Angsd v0.928 (55) was used to calculate genotype likelihoods for each individual and to estimate allele frequencies in each vulnerability group for both densities. The following site filtering options were used in ANGSD: -SNP_pval 1e-6 -remove_bads 1 (removal of bad mapped reads), -setMinDepth 48 (minimum sum of depth across individuals), -setMaxDepth 600 (maximum sum of depth across individuals), -minInd 48 (minimum number of individuals), -minQ 20 (minimum read quality), -minMapQ 20 (minimum mapping quality), and -minMaf 0.05 (minimum minor allele frequency). We used the group allele frequencies from ANGSD to calculate z-transformed differences in allele frequencies (zdAF) between the captured and escaped fish in each density [i.e., zdAF = dAF – mean(dAF)/sd(dAF)] using R v3.5.1 (56). For additional analyses, we also subdivided the fish from each density and vulnerability group into two post hoc groups with family shared evenly, to create two replicated genomic analyses within each density. A global maximum likelihood estimate of expected heterozygozity (H_e_) was calculated in ANGSD using the real site frequency spectrum (SFS) function on all the allele frequencies.

#### Delta allele frequency outlier detection, functional enrichment analysis, and analysis of SNP types.

To determine the outlier threshold, we created 25 permutation groups of 24 randomly chosen individuals, inferred group allele frequencies in ANGSD using the same data filtering options as described in the previous paragraph, and recalculated zdAF between the 625 possible pairings of 25 permutation groups. We thus estimated the random zdAF distribution for each SNP position derived from the 625 zdAF values obtained from the different pairings. From this random zdAF distribution per position, we first considered the upper and lower 0.05% quantiles after Bonferroni correction for the total number of SNPs (*P* < 8.8e-11, i.e., 0.0005/5.67M; equivalent to Bonferroni-corrected empirical *P* values, using “function (x) quantile (x, *p*-value)” in R) to be the significant zdAF threshold at each SNP. This analysis generated a distribution of SNP thresholds across the genome that we then compiled and used to calculate a “global” 0.5% quantile threshold. Outlier SNPs between captured and escaped fish in each density were then defined as those with zdAF values exceeding the upper or lower global 0.5% quantiles of the 0.05% Bonferroni-corrected quantile thresholds.

Outlier SNPs were annotated using the annotations contained in the zebrafish reference genome (GRCz11) RefSeq annotation file available on National Center for Biotechnology Information (NCBI). Using the annotated outlier SNPs, we performed a PANTHER-based GO term enrichment analysis (57) on the Gene Ontology webpage (http://geneontology.org/) (58), applying a significance threshold of FDR < 0.05. In addition, using the total set of SNPs, we averaged the zdAF values of each gene in each density population, and performed a gene set enrichment analysis in WebGestalt (59–61), applying again a significance threshold of FDR < 0.05. The total set of SNPs and the outlier SNPs from each density were also used for an analysis of SNP types using SnpEff v4.4 (62). We first created a SnpEff database based on the GCA_000002035.4_GRCz11_genomic.fna sequence file and the GCF_000002035.6_GRCz11_genomic.gff annotation file available on NCBI. The chromosome name format in the annotation file was changed to the format in the sequence file. Then, we analyzed SNP types using SnpEff on each set of SNPs.

#### PCA.

PCAngsd v0.98 (63) was used to obtain PC scores based on the BEAGLE genotype likelihood files from ANGSD using only sites of zdAF outlier SNPs. Based on the separation of the captured and escaped individuals in the PCA plots, the PC2 scores were used for subsequent statistical analysis, as PC1 mainly clustered the variability within the escaped fish from the reduced density.

### Statistics.

Data normality and homogeneity of variance were tested according to the analysis of the distribution of model residuals and Levene tests respectively. The level of aggressiveness (number of bites against a mirror) and the PC score based on allele frequency difference of the outlier SNPs were not normally distributed, so data were ranked, and statistical procedures were applied on ranks (64). A general linear model multivariate analysis of covariance was used to analyze the global shift of the fish phenotypes (including fish SGR, AS, level of aggressiveness, and sociability) with sex, density, and vulnerability as well as their interaction as fixed effects and mass as a covariate. Subsequently, a general linear model was used to analyze the fish individual phenotypes SGR, AS, level of aggressiveness, and sociability with sex, density, and vulnerability as well as their interaction, fitted as fixed effects, and mass (or length in the case of sociability) as a covariate. Tank effects on the phenotypic variables were not significant (GLM: growth rate, F3,282 = 2.09, *P* = 0.11; aerobic scope, F3,282 = 0.55, *P* = 0.65; aggression, F3,259 = 2.07, *P* = 0.11; sociability, F3,256 = 0.05, *P* = 0.98). The PC score was also analyzed using a similar general linear model but without covariate. A posteriori Tukey tests were used for mean comparisons. The correlation between the PC score and mass, SGR, AS, level of aggressiveness, and sociability was evaluated using the Pearson correlation coefficient. Statistical analyses were performed using Statistica 7 (Statsoft), and all visuals were created using ggplot2 in R v3.5.3. A significance level of α = 0.05 was used in all statistical tests.

## Supplementary Material

Supplementary File

## Data Availability

All behavioral, physiological, and genomic data collected for the study are openly available in the University of Glasgow Enlighten at http://dx.doi.org/10.5525/gla.researchdata.1011 (65) and BioProject ID PRJNA630223 on NCBI (66).
