# Supplementary material for: Genomic basis of fishing-associated selection varies with population density
Source: Proc Natl Acad Sci U S A. 2021 Dec 13;118(51):e2020833118. doi: 10.1073/pnas.2020833118 (PMC8713780; doi:10.1073/pnas.2020833118)
Supplement: Supplementary File [file pnas.2020833118.sapp.pdf]

PNAS
www.pnas.org

Supplementary Information for

Genomic basis of fishing-associated selection varies with population density.

Amélie Crespel1,2*, Kevin Schneider1, Toby Miller1, Anita Rácz1,3, Arne Jacobs1,4, Jan Lindström1, Kathryn R. Elmer1, Shaun S. Killen1*
Email:amelie.crespel@gmail.com, shaun.killen@glasgow.ac.uk

This PDF file includes:

Supplementary text
Figures S1 to S4
Tables S1 to S6

Supplementary Information Text

Replication analysis within experimental groups.

As a confirmation that between-density differences were greater than within-density differences, we examined the difference in allele frequency between two random post-hoc groups of 12 fish within each density using a new genotype likelihood analysis. Using the same threshold as before, we identified 1781 and 1531 outlier SNPs that differed between the captured and escaped fish in the two post-hoc groups within the baseline density population, and 1405 and 1688 outlier SNPs in the two post-hoc groups within the reduced density population. From the genes identified with these outliers SNPs, 114 were shared between the two post-hoc groups in the baseline density and 132 were shared between the two post-hoc groups in the reduced density, which was greater than expected by chance (Fisher's Exact Test, $P < 0.0001$ for both) (Table S3). Only six genes were shared across the four groups (similarly to what could be expected by chance, Fisher's Exact Test, $P = 0.07$). All the genes identified were again mainly involved in brain function and neurogenesis (Table S4). A multivariate analysis (principal component analysis, PCA) was also conducted using the two random post-hoc groups within each density and no difference was observed in the genomic PC scores between the two post hoc replicates (Figure S3). In addition, a similar multivariate analysis was conducted using the different rearing tanks as replicates within each density. Again, no difference was observed across the tank replicates within density (Figure S4).

A

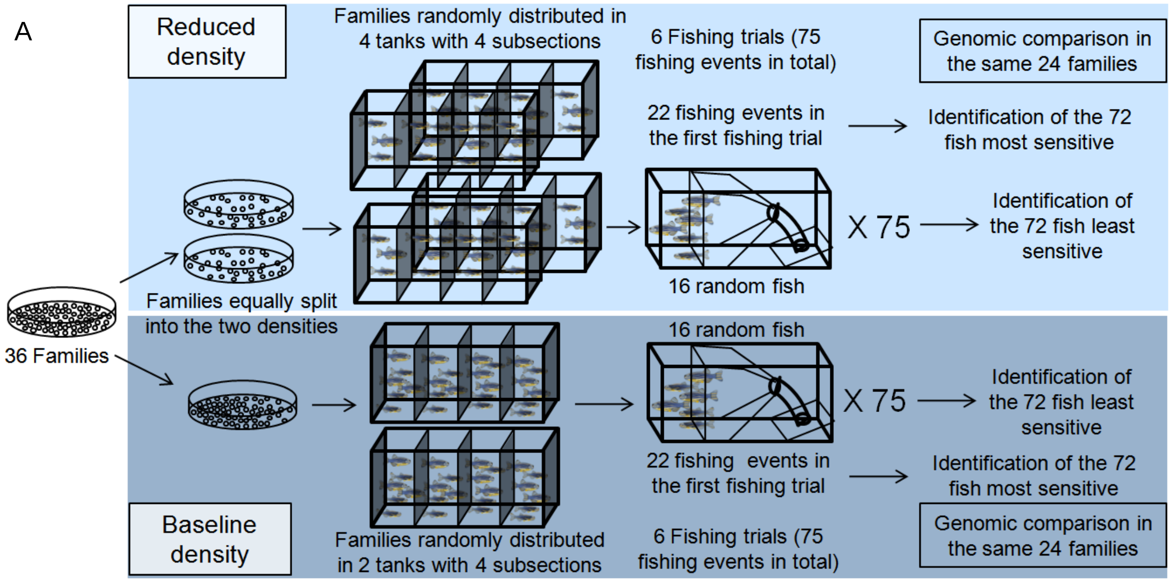

B

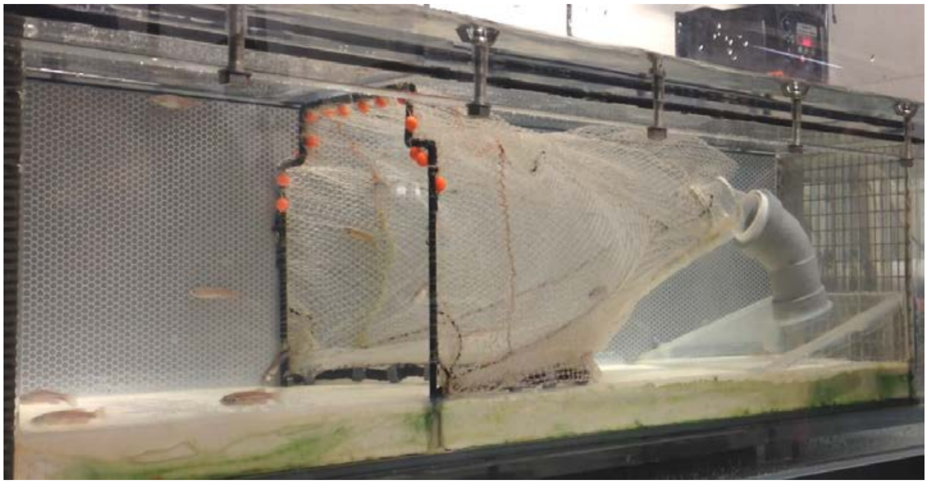

Fig. S1. Design of our experiment and trawling simulations. A) Details of our experimental design. We created 36 families that were equally split at hatching into two densities (baseline density or reduced density, i.e. half of baseline density). At 6 months, 10 fish per family and density were individually tagged and randomly distributed and housed in multiple 55L tanks (four tanks for the reduced density and two tanks for the baseline density) subdivided into four equal sections. The trawling selection was then conducted over a number of fishing trials (six in total), each trial consisting of several individual fishing events conducted on 16 fish randomly selected from each density. In the first fishing trial, 22 fishing events were conducted (a total of 75 fishing events were conducted over the six fishing trials). At the end of a fishing trial, the 20% of the fish

the most vulnerable in each density were identified according to their time of capture and removed from the subsequent trials. The other fish were returned to the housing subsections randomly. The first fishing trial allowed the identification of the 20% of fish the most vulnerable (N = 72 per density, considered as our captured groups) while at the end of the six fishing trials, the 20% of fish the least vulnerable (N=72 per density, considered as our escaped group) were identified as escaped every trawling simulation. We sequenced 24 siblings from the same family origin across the four groups (each density/vulnerability group) to investigate genomic difference. B) Photo of the trawling simulation on zebrafish. The simulations took place in a 90L swimming tunnel (Loligo Systems, Denmark) using a small-scale model trawl completed by an acrylic compartment to protect the fish from being compressed by the flow (designed by the Fisheries and Marine Institute of Memorial University of Newfoundland).

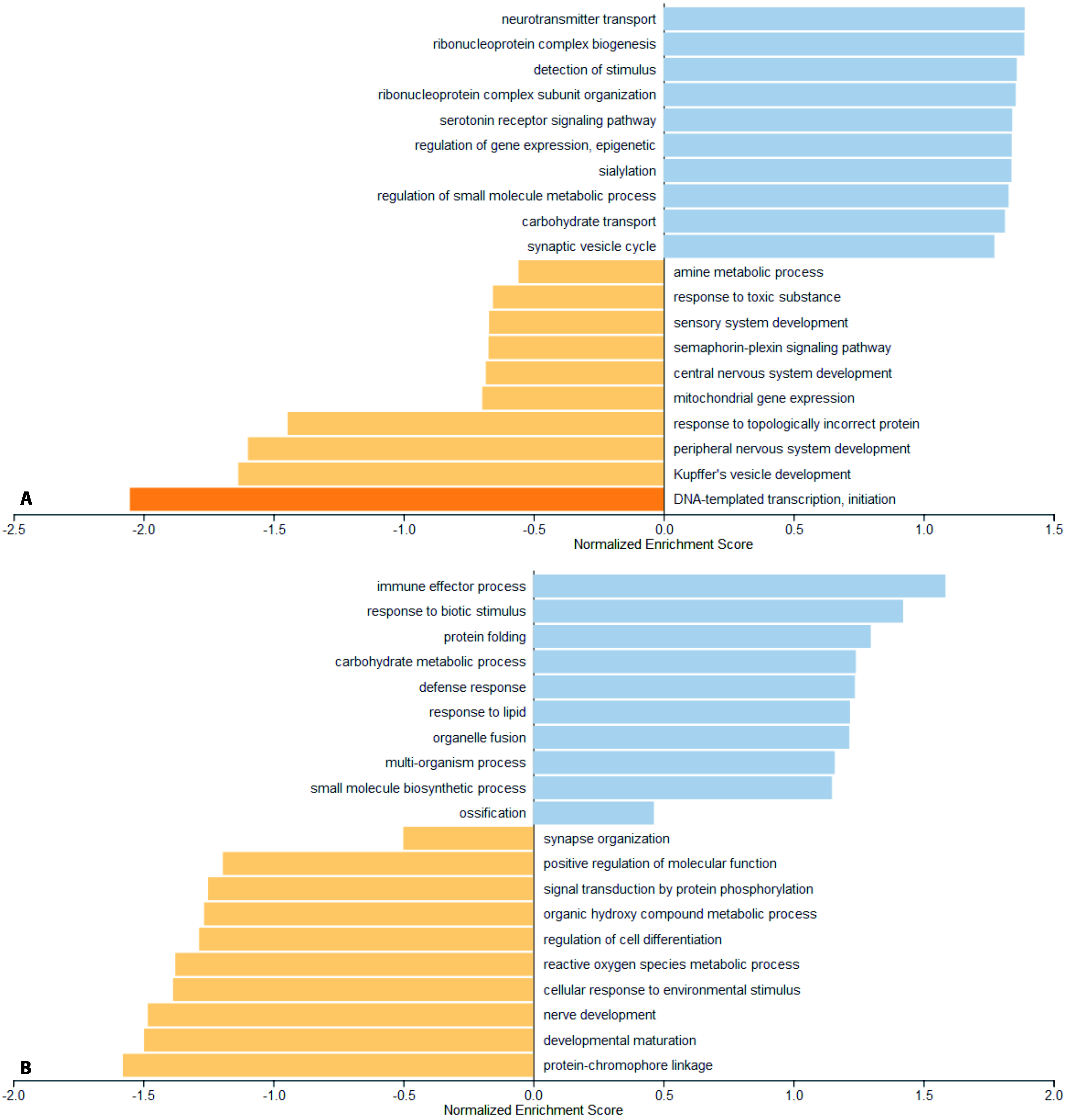
A
neurotransmitter transport
ribonucleoprotein complex biogenesis
detection of stimulus
ribonucleoprotein complex subunit organization
serotonin receptor signaling pathway
regulation of gene expression, epigenetic
sialylation
regulation of small molecule metabolic process
carbohydrate transport
synaptic vesicle cycle
amine metabolic process
response to toxic substance
sensory system development
semaphorin-plexin signaling pathway
central nervous system development
mitochondrial gene expression
response to topologically incorrect protein
peripheral nervous system development
Kupffer's vesicle development
DNA-templated transcription, initiation
-2.5
-2.0
-1.5
-1.0
-0.5
0.0
0.5
1.0
1.5
Normalized Enrichment Score
B
immune effector process
response to biotic stimulus
protein folding
carbohydrate metabolic process
defense response
response to lipid
organelle fusion
multi-organism process
small molecule biosynthetic process
ossification
synapse organization
positive regulation of molecular function
signal transduction by protein phosphorylation
organic hydroxy compound metabolic process
regulation of cell differentiation
reactive oxygen species metabolic process
cellular response to environmental stimulus
nerve development
developmental maturation
protein-chromophore linkage
-2.0
-1.5
-1.0
-0.5
0.0
0.5
1.0
1.5
2.0
Normalized Enrichment Score

Fig. S2. GO terms with shifts in z-transformed differences in allele frequencies, i.e. zdAF, in the two density populations (Gene set enrichment analysis). Significance and fold enrichment (Normalized Enrichment Score) of the 10 most enriched GO terms with positive (blue) and negative (yellow) zdAF shifts using the total set of SNPs in the fish reared under a baseline (A) or reduced (B) density. Darker bars are the GO terms with significant enrichment (FDR < 0.05).

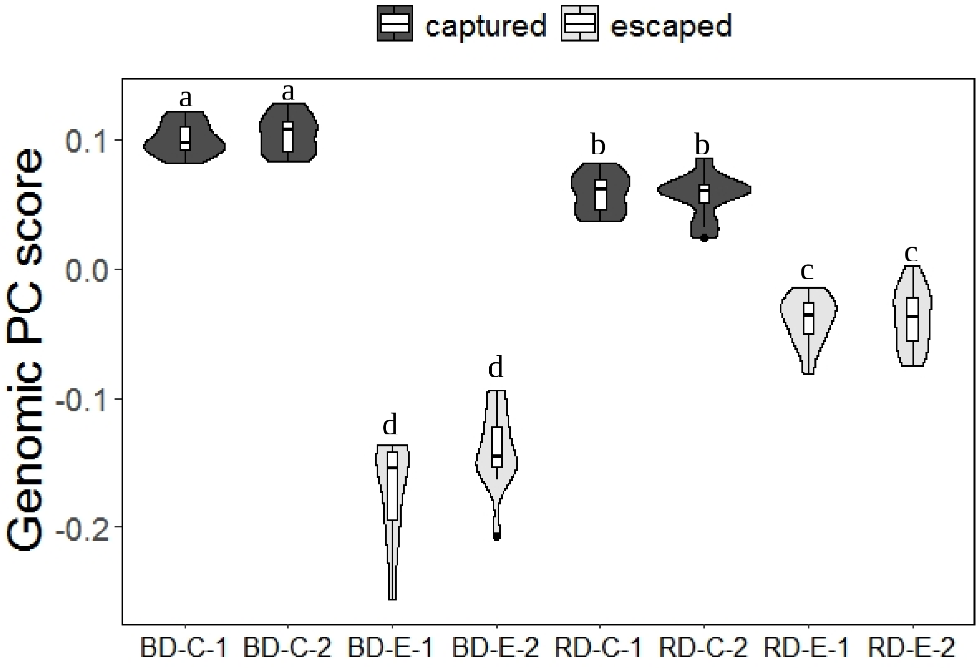

Fig. S3. Genomic selection in the two post-hoc groups of each density populations. Distribution of the genomic PC score of the outliers of captured (C) (dark grey) and escaped fish (E) (light grey) after a series of trawling simulations, for the different groups reared either under a baseline (BD) or reduced (RD) density (N = 12 per group). Different letters indicate significant difference among the conditions (GLM: $P < 0.05$). There was no difference in genomic PC scores between the two post hoc replicates.

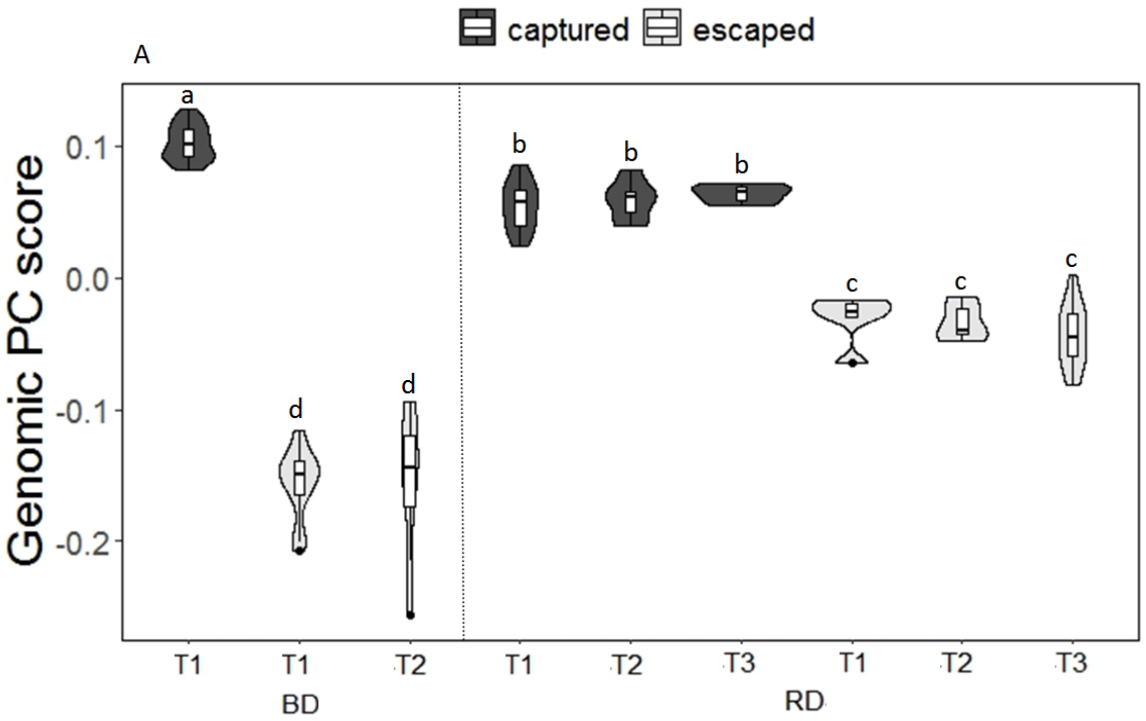

Fig. S4. Genomic selection in the rearing tanks of each density populations. Distribution of the genomic PC score of the outliers of captured (C) (dark grey) and escaped fish (E) (light grey) after a series of trawling simulations, for the different groups reared either under a baseline (BD) or reduced (RD) density. T represents the tank number. Tanks not represented means that no fish from the group were genotyped in these tanks. Different letters indicate significant difference among the conditions (GLM: $P < 0.05$). There was no difference in genomic PC scores between the tanks replicates within each vulnerability category and treatment combination.

Table S1. Complete list of the outliers GO terms of the genes selected by the fishing simulation in the two density populations. Significance (P-values and FDR) and fold enrichment of the GO terms represented in the outliers of the fish reared under a baseline or reduced density. GO is the Gene Ontology ID for each biological process. Grey GO terms represent GO terms shared between the two populations, bold GO terms are the GO terms with significant enrichment (FDR < 0.05).

GO biological process	*Danio rerio* - Reference	Number of genes	Expected	Fold Enrichment	P-values	FDR values
**Baseline density**						
nervous system development (GO:0007399)	1719	26	11.84	2.2	2.04E-04	4.12E-01
generation of neurons (GO:0048699)	980	16	6.75	2.37	1.40E-03	5.42E-01
brain morphogenesis (GO:0048854)	30	3	0.21	14.52	1.48E-03	5.54E-01
retina morphogenesis in camera-type eye (GO:0060042)	124	5	0.85	5.85	1.97E-03	5.79E-01
cell-cell adhesion via plasma-membrane adhesion molecules (GO:0098742)	202	6	1.39	4.31	3.16E-03	6.25E-01
neuron development (GO:0048666)	716	12	4.93	2.43	4.48E-03	6.59E-01
neurogenesis (GO:0022008)	1099	16	7.57	2.11	4.66E-03	6.93E-01
actin filament organization (GO:0007015)	294	7	2.02	3.46	4.81E-03	7.27E-01
regulation of actin filament depolymerization (GO:0030834)	48	3	0.33	9.07	5.15E-03	7.39E-01
actin filament-based process (GO:0030029)	548	10	3.77	2.65	5.23E-03	7.54E-01
neuron differentiation (GO:0030182)	893	14	6.15	2.28	5.71E-03	8.05E-01
dendrite self-avoidance (GO:0070593)	15	2	0.1	19.36	5.91E-03	8.25E-01
cell-cell adhesion (GO:0098609)	307	7	2.11	3.31	6.02E-03	8.34E-01
actin filament bundle organization (GO:0061572)	51	3	0.35	8.54	6.04E-03	8.42E-01

homophilic cell adhesion via plasma membrane adhesion molecules (GO:0007156)	166	5	1.14	4.37	6.53E-03	8.59E-01
cytoskeleton organization (GO:0007010)	933	14	6.43	2.18	7.05E-03	8.72E-01
behavior (GO:0007610)	171	5	1.18	4.25	7.36E-03	9.11E-01
respiratory system development (GO:0060541)	17	2	0.12	17.08	7.37E-03	9.13E-01
camera-type eye morphogenesis (GO:0048593)	178	5	1.23	4.08	8.63E-03	9.50E-01
optokinetic behavior (GO:0007634)	19	2	0.13	15.28	8.97E-03	9.51E-01
**Reduced density**						
**nervous system development (GO:0007399)**	**1719**	**31**	**13.38**	**2.32**	**1.35E-05**	**4.18E-02**
**neuron development (GO:0048666)**	**716**	**19**	**5.57**	**3.41**	**4.72E-06**	**4.39E-02**
**multicellular organismal process (GO:0032501)**	**4937**	**63**	**38.42**	**1.64**	**4.31E-05**	**4.46E-02**
**multicellular organism development (GO:0007275)**	**4095**	**55**	**31.87**	**1.73**	**3.89E-05**	**4.52E-02**
**plasma membrane bounded cell projection organization (GO:0120036)**	**871**	**20**	**6.78**	**2.95**	**2.07E-05**	**4.81E-02**
**cell projection organization (GO:0030030)**	**902**	**21**	**7.02**	**2.99**	**1.04E-05**	**4.83E-02**
system development (GO:0048731)	3610	50	28.1	1.78	3.86E-05	5.13E-02
neurogenesis (GO:0022008)	1099	22	8.55	2.57	5.98E-05	5.56E-02
neuron projection development (GO:0031175)	550	15	4.28	3.5	3.62E-05	5.61E-02
generation of neurons (GO:0048699)	980	21	7.63	2.75	3.44E-05	6.39E-02
protein localization to cell junction (GO:1902414)	29	4	0.23	17.72	1.19E-04	7.35E-02

neuron projection guidance (GO:0097485)	287	10	2.23	4.48	1.12E-04	7.44E-02
neuron differentiation (GO:0030182)	893	19	6.95	2.73	9.04E-05	7.64E-02
brain morphogenesis (GO:0048854)	30	4	0.23	17.13	1.33E-04	7.76E-02
axon guidance (GO:0007411)	286	10	2.23	4.49	1.09E-04	7.79E-02
axonogenesis (GO:0007409)	405	12	3.15	3.81	1.05E-04	8.10E-02
axon development (GO:0061564)	426	12	3.32	3.62	1.65E-04	8.53E-02
cell development (GO:0048468)	1354	24	10.54	2.28	1.89E-04	8.77E-02
locomotion (GO:0040011)	935	19	7.28	2.61	1.61E-04	8.83E-02
cell morphogenesis involved in neuron differentiation (GO:0048667)	431	12	3.35	3.58	1.83E-04	8.97E-02
chemotaxis (GO:0006935)	436	12	3.39	3.54	2.03E-04	9.00E-02
cellular component organization (GO:0016043)	3858	50	30.03	1.67	2.20E-04	9.29E-02
animal organ development (GO:0048513)	2595	37	20.2	1.83	3.50E-04	1.05E-01
protein localization to postsynaptic membrane (GO:1903539)	15	3	0.12	25.7	3.40E-04	1.05E-01
cell part morphogenesis (GO:0032990)	466	12	3.63	3.31	3.65E-04	1.06E-01
cellular process (GO:0009987)	14878	141	115.79	1.22	3.04E-04	1.09E-01
protein localization to postsynapse (GO:0062237)	15	3	0.12	25.7	3.40E-04	1.09E-01
anatomical structure development (GO:0048856)	4438	55	34.54	1.59	3.31E-04	1.10E-01
cellular component organization or biogenesis (GO:0071840)	4034	51	31.4	1.62	4.03E-04	1.10E-01
cell projection morphogenesis (GO:0048858)	459	12	3.57	3.36	3.20E-04	1.10E-01
plasma membrane bounded cell projection morphogenesis (GO:0120039)	455	12	3.54	3.39	2.96E-04	1.10E-01
central nervous system development	688	15	5.35	2.8	3.94E-04	1.11E-01

(GO:0007417)						
neuron projection morphogenesis (GO:0048812)	455	12	3.54	3.39	2.96E-04	1.15E-01
taxis (GO:0042330)	453	12	3.53	3.4	2.85E-04	1.15E-01
receptor localization to synapse (GO:0097120)	43	4	0.33	11.95	4.75E-04	1.26E-01
brain development (GO:0007420)	486	12	3.78	3.17	5.26E-04	1.36E-01
cellular component morphogenesis (GO:0032989)	562	13	4.37	2.97	5.64E-04	1.42E-01
protein localization to synapse (GO:0035418)	19	3	0.15	20.29	6.27E-04	1.53E-01
movement of cell or subcellular component (GO:0006928)	1120	20	8.72	2.29	6.77E-04	1.61E-01
head development (GO:0060322)	505	12	3.93	3.05	7.29E-04	1.70E-01
regulation of biological quality (GO:0065008)	2008	30	15.63	1.92	8.02E-04	1.73E-01
cell population proliferation (GO:0008283)	134	6	1.04	5.75	7.83E-04	1.73E-01
cell-cell adhesion (GO:0098609)	307	9	2.39	3.77	8.24E-04	1.74E-01
cell morphogenesis involved in differentiation (GO:0000904)	509	12	3.96	3.03	7.80E-04	1.77E-01
cell morphogenesis (GO:0000902)	592	13	4.61	2.82	8.99E-04	1.86E-01
anatomical structure morphogenesis (GO:0009653)	2091	30	16.27	1.84	1.10E-03	2.23E-01
ephrin receptor signaling pathway (GO:0048013)	24	3	0.19	16.06	1.16E-03	2.24E-01
developmental process (GO:0032502)	4625	55	36	1.53	1.15E-03	2.27E-01
axon midline choice point recognition (GO:0016199)	6	2	0.05	42.83	1.61E-03	2.88E-01
sensory organ development (GO:0007423)	709	14	5.52	2.54	1.55E-03	2.94E-01
forebrain morphogenesis (GO:0048853)	6	2	0.05	42.83	1.61E-03	2.94E-01

localization (GO:0051179)	3970	48	30.9	1.55	1.60E-03	2.98E-01
ventricular system development (GO:0021591)	28	3	0.22	13.77	1.74E-03	3.05E-01
epithelial cell-cell adhesion (GO:0090136)	7	2	0.05	36.71	2.06E-03	3.55E-01
axon choice point recognition (GO:0016198)	8	2	0.06	32.12	2.56E-03	4.04E-01
magnesium ion homeostasis (GO:0010960)	8	2	0.06	32.12	2.56E-03	4.11E-01
metal ion transport (GO:0030001)	229	7	1.78	3.93	2.47E-03	4.18E-01
protein K11-linked ubiquitination (GO:0070979)	8	2	0.06	32.12	2.56E-03	4.18E-01
fourth ventricle development (GO:0021592)	8	2	0.06	32.12	2.56E-03	4.26E-01
animal organ morphogenesis (GO:0009887)	828	15	6.44	2.33	3.71E-03	5.74E-01
metal ion homeostasis (GO:0055065)	388	9	3.02	2.98	3.87E-03	5.90E-01
homeostatic process (GO:0042592)	851	15	6.62	2.26	4.18E-03	6.27E-01
establishment of protein localization to postsynaptic membrane (GO:1903540)	12	2	0.09	21.41	5.08E-03	7.16E-01
cellular metal ion homeostasis (GO:0006875)	330	8	2.57	3.11	4.92E-03	7.27E-01
neurotransmitter receptor transport to plasma membrane (GO:0098877)	12	2	0.09	21.41	5.08E-03	7.27E-01
neurotransmitter receptor transport to postsynaptic membrane (GO:0098969)	12	2	0.09	21.41	5.08E-03	7.38E-01
chemical homeostasis (GO:0048878)	567	11	4.41	2.49	5.50E-03	7.63E-01
inner ear morphogenesis (GO:0042472)	89	4	0.69	5.77	5.90E-03	8.07E-01
ear morphogenesis (GO:0042471)	90	4	0.7	5.71	6.13E-03	8.26E-01
cell differentiation (GO:0030154)	2327	30	18.11	1.66	6.23E-03	8.28E-01
immune system process (GO:0002376)	1232	2	9.59	0.21	6.79E-03	8.42E-01
regulation of postsynaptic membrane neurotransmitter receptor levels (GO:0099072)	46	3	0.36	8.38	6.44E-03	8.43E-01

developmental growth (GO:0048589)	277	7	2.16	3.25	6.74E-03	8.48E-01
cellular developmental process (GO:0048869)	2346	30	18.26	1.64	6.60E-03	8.52E-01
growth (GO:0040007)	277	7	2.16	3.25	6.74E-03	8.59E-01
embryonic organ morphogenesis (GO:0048562)	427	9	3.32	2.71	7.03E-03	8.61E-01
cellular potassium ion homeostasis (GO:0030007)	15	2	0.12	17.13	7.48E-03	8.92E-01
cytoskeleton organization (GO:0007010)	933	15	7.26	2.07	7.58E-03	8.92E-01
regulation of secretion by cell (GO:1903530)	153	5	1.19	4.2	7.77E-03	9.03E-01
sodium ion export across plasma membrane (GO:0036376)	15	2	0.12	17.13	7.48E-03	9.03E-01
otic vesicle development (GO:0071599)	51	3	0.4	7.56	8.43E-03	9.11E-01
cation homeostasis (GO:0055080)	441	9	3.43	2.62	8.56E-03	9.15E-01
cell migration involved in heart formation (GO:0060974)	16	2	0.12	16.06	8.37E-03	9.16E-01
dorsal convergence (GO:0060030)	16	2	0.12	16.06	8.37E-03	9.27E-01
regulation of secretion (GO:0051046)	158	5	1.23	4.07	8.82E-03	9.32E-01
chordate embryonic development (GO:0043009)	517	10	4.02	2.49	8.13E-03	9.33E-01
sensory organ morphogenesis (GO:0090596)	299	7	2.33	3.01	9.94E-03	9.33E-01
cellular sodium ion homeostasis (GO:0006883)	16	2	0.12	16.06	8.37E-03	9.38E-01
inorganic ion homeostasis (GO:0098771)	451	9	3.51	2.56	9.79E-03	9.38E-01
actin cytoskeleton organization (GO:0030036)	533	10	4.15	2.41	9.89E-03	9.39E-01
inorganic ion transmembrane transport (GO:0098660)	526	10	4.09	2.44	9.09E-03	9.39E-01

anaphase-promoting complex-dependent catabolic process (GO:0031145)	17	2	0.13	15.12	9.31E-03	9.41E-01
divalent inorganic cation homeostasis (GO:0072507)	296	7	2.3	3.04	9.45E-03	9.45E-01
embryo development ending in birth or egg hatching (GO:0009792)	519	10	4.04	2.48	8.33E-03	9.45E-01
visual system development (GO:0150063)	532	10	4.14	2.42	9.77E-03	9.47E-01
biological regulation (GO:0065007)	10581	101	82.35	1.23	9.27E-03	9.48E-01
embryonic organ development (GO:0048568)	609	11	4.74	2.32	9.07E-03	9.48E-01
cellular homeostasis (GO:0019725)	456	9	3.55	2.54	1.05E-02	9.53E-01
cellular cation homeostasis (GO:0030003)	378	8	2.94	2.72	1.06E-02	9.53E-01
neural tube patterning (GO:0021532)	55	3	0.43	7.01	1.03E-02	9.53E-01
eye development (GO:0001654)	532	10	4.14	2.42	9.77E-03	9.57E-01
homophilic cell adhesion via plasma membrane adhesion molecules (GO:0007156)	166	5	1.29	3.87	1.07E-02	9.57E-01
export from cell (GO:0140352)	227	6	1.77	3.4	9.68E-03	9.57E-01
gene expression (GO:0010467)	1358	3	10.57	0.28	1.04E-02	9.59E-01

Table S2. List and GO terms of the eight outlier genes selected by the fishing simulation in both the baseline and reduced density. (A) List of the eight outlier genes (B) Significance (P-values and FDR) and fold enrichment of the GO terms represented in the outlier genes shared between the fish reared under a baseline or reduced density. GO is the Gene Ontology ID for each biological process.

A List of the outlier genes

SNPs overlapping between density	Accession Number	Abbreviation	Gene name
overlapping SNP	ENSDARG00000099673	si:dkey-203a12.5	Si:dkey-203a12.5
non-overlapping SNP(s)	ENSDARG00000015567	zic1	Odd-paired-like
non-overlapping SNP(s)	ENSDARG00000024785	ctnna2	Catenin (cadherin-associated protein), alpha 2
non-overlapping SNP(s)	ENSDARG00000043332	prkcz	Protein kinase C
non-overlapping SNP(s)	ENSDARG00000044574	rbms3	RNA-binding motif, single-stranded-interacting protein 3
non-overlapping SNP(s)	ENSDARG00000052644	ca10a	Carbonic anhydrase Xa
non-overlapping SNP(s)	ENSDARG00000059846	epg5	Ectopic P granules protein 5 homolog
non-overlapping SNP(s)	ENSDARG00000086034	nectin1b	Nectin 1b

B GO terms of the outlier genes

GO biological process	*Danio rerio* - Reference	Number of genes	Expected	Fold Enrichment	P-values	FDR values
brain morphogenesis (GO:0048854)	34	2	0.01	> 100	3.93E-05	1.76E-01
regulation of synapse structure or activity (GO:0050803)	53	2	0.01	> 100	9.24E-05	2.76E-01
regulation of synapse organization (GO:0050807)	32	2	0.01	> 100	3.50E-05	3.14E-01
camera-type eye morphogenesis (GO:0048593)	164	2	0.04	45.1	8.40E-04	6.28E-01

animal organ morphogenesis (GO:0009887)	796	3	0.22	13.94	9.33E-04	6.44E-01
adhesion of symbiont to host (GO:0044406)	2	1	0	> 100	8.11E-04	6.62E-01
adhesion of symbiont to host cell (GO:0044650)	2	1	0	> 100	8.11E-04	7.28E-01
regulation of developmental process (GO:0050793)	861	3	0.23	12.89	1.17E-03	7.51E-01
retina development in camera-type eye (GO:0060041)	219	2	0.06	33.77	1.48E-03	7.82E-01
eye morphogenesis (GO:0048592)	206	2	0.06	35.91	1.31E-03	7.86E-01
cell proliferation in hindbrain (GO:0021534)	5	1	0	> 100	1.62E-03	8.08E-01
virion attachment to host cell (GO:0019062)	2	1	0	> 100	8.11E-04	8.09E-01
regulation of multicellular organismal process (GO:0051239)	927	3	0.25	11.97	1.45E-03	8.13E-01
cell differentiation involved in pronephros development (GO:0039014)	7	1	0	> 100	2.16E-03	8.43E-01
forebrain morphogenesis (GO:0048853)	6	1	0	> 100	1.89E-03	8.49E-01
fourth ventricle development (GO:0021592)	8	1	0	> 100	2.43E-03	8.73E-01
nephron tubule epithelial cell differentiation (GO:0072160)	7	1	0	> 100	2.16E-03	8.82E-01
cell-cell adhesion (GO:0098609)	247	2	0.07	29.95	1.87E-03	8.85E-01
sensory organ morphogenesis (GO:0090596)	282	2	0.08	26.23	2.43E-03	9.08E-01
dermal bone morphogenesis	2	1	0	> 100	8.11E-04	9.10E-01

(GO:0061972)						
pronephric nephron tubule epithelial cell differentiation (GO:0035778)	7	1	0	> 100	2.16E-03	9.23E-01
regulation of multicellular organismal development (GO:2000026)	692	3	0.19	16.03	6.22E-04	9.30E-01
retina morphogenesis in camera-type eye (GO:0060042)	115	2	0.03	64.32	4.19E-04	9.40E-01
regulation of synapse structural plasticity (GO:0051823)	1	1	0	> 100	5.41E-04	9.70E-01

Table S3. Summary table of the genes identified as outliers after the fishing simulation in the two post-hoc replicated groups of each population density. Fishing selection impact on the annotated genes, gene ontology (GO) terms and enriched gene ontology (GO) terms in the two random sub-groups of the baseline density and reduced density. The list of the GO terms enriched from each population density is available in the Table S4. From the annotated genes identified, asterisks in the shared groups indicate higher than expected by chance (Fisher's Exact Test, $P < 0.0001$, the total number of annotated genes in the zebrafish genome was 32057 at the time of analysis).

	Within Baseline density			Within Reduced density			Between densities
	Group 1	Group 2	Shared	Group 1	Group 2	Shared	Shared
Annotated genes	873	787	114*	657	849	132*	6
GO terms	188	84	55	218	348	123	45
GO terms enriched	23	7	5	27	102	22	5

Table S4. List of the enriched GO terms of the outlier genes selected by the fishing simulation in the two groups of each density populations. Significance (P-values and FDR) and fold enrichment of the GO terms represented in the outliers of the fish reared under a baseline or reduced density. GO is the Gene Ontology ID for each biological process. Grey GO terms represent GO terms shared between the two groups in each population, bold GO terms are the GO terms shared in the four groups.

GO biological process	*Danio rerio* - Reference	Number of genes	Expected	Fold Enrichment	P-values	FDR values
<u>**Baseline density group 1**</u>						
cell-cell adhesion (GO:0098609)	302	43	8.78	4.9	3.60E-16	3.34E-12
**biological adhesion (GO:0022610)**	651	62	18.92	3.28	4.78E-15	1.48E-11
**cell adhesion (GO:0007155)**	651	62	18.92	3.28	4.78E-15	2.22E-11
cell-cell adhesion via plasma-membrane adhesion molecules (GO:0098742)	196	30	5.7	5.27	2.18E-12	5.05E-09
homophilic cell adhesion via plasma membrane adhesion molecules (GO:0007156)	166	26	4.83	5.39	4.08E-11	7.57E-08
**cellular process (GO:0009987)**	14851	510	431.69	1.18	7.72E-09	1.19E-05
movement of cell or subcellular component (GO:0006928)	1116	64	32.44	1.97	8.34E-07	1.11E-03
regulation of trans-synaptic signaling (GO:0099177)	175	19	5.09	3.74	2.82E-06	2.91E-03
modulation of chemical synaptic transmission (GO:0050804)	175	19	5.09	3.74	2.82E-06	3.28E-03
locomotion (GO:0040011)	927	54	26.95	2	4.45E-06	4.13E-03
biological_process (GO:0008150)	19556	620	568.46	1.09	6.69E-06	5.17E-03
multicellular organism development (GO:0007275)	4073	166	118.4	1.4	7.89E-06	5.64E-03
Unclassified (UNCLASSIFIED)	6142	127	178.54	0.71	6.69E-06	5.64E-03

anatomical structure development (GO:0048856)	4410	174	128.19	1.36	2.91E-05	1.93E-02
**developmental process (GO:0032502)**	4603	179	133.8	1.34	4.02E-05	2.49E-02
**nervous system development (GO:0007399)**	1707	80	49.62	1.61	4.33E-05	2.51E-02
system development (GO:0048731)	3586	145	104.24	1.39	4.87E-05	2.66E-02
cell motility (GO:0048870)	690	40	20.06	1.99	9.02E-05	3.81E-02
multicellular organismal process (GO:0032501)	4925	188	143.16	1.31	7.59E-05	3.91E-02
cell migration (GO:0016477)	644	38	18.72	2.03	8.49E-05	3.94E-02
calcium-ion regulated exocytosis (GO:0017156)	44	8	1.28	6.25	9.79E-05	3.95E-02
localization of cell (GO:0051674)	690	40	20.06	1.99	9.02E-05	3.99E-02
neuron cell-cell adhesion (GO:0007158)	12	5	0.35	14.33	8.28E-05	4.04E-02
**Baseline density group 2**						
**biological adhesion (GO:0022610)**	651	40	17.02	2.35	1.78E-06	8.27E-03
cellular component organization or biogenesis (GO:0071840)	4026	151	105.28	1.43	5.00E-06	9.28E-03
cellular component organization (GO:0016043)	3851	146	100.7	1.45	4.34E-06	1.01E-02
**nervous system development (GO:0007399)**	1707	78	44.64	1.75	3.58E-06	1.11E-02
**cell adhesion (GO:0007155)**	651	40	17.02	2.35	1.78E-06	1.65E-02
**cellular process (GO:0009987)**	14851	444	388.35	1.14	1.54E-05	2.39E-02
**developmental process (GO:0032502)**	4603	165	120.37	1.37	2.36E-05	3.12E-02
**Reduced density group 1**						
cell-cell adhesion (GO:0098609)	302	25	6.53	3.83	3.97E-08	1.23E-04
**cellular process (GO:0009987)**	14851	384	321.32	1.2	6.54E-08	1.52E-04

**biological adhesion (GO:0022610)**	651	39	14.08	2.77	3.39E-08	1.58E-04
system development (GO:0048731)	3586	124	77.59	1.6	1.42E-07	2.63E-04
**cell adhesion (GO:0007155)**	651	39	14.08	2.77	3.39E-08	3.15E-04
multicellular organism development (GO:0007275)	4073	135	88.12	1.53	4.27E-07	6.60E-04
multicellular organismal process (GO:0032501)	4925	156	106.56	1.46	5.27E-07	6.99E-04
cellular developmental process (GO:0048869)	2354	88	50.93	1.73	8.71E-07	1.01E-03
cell differentiation (GO:0030154)	2335	87	50.52	1.72	1.17E-06	1.20E-03
Unclassified (UNCLASSIFIED)	6142	86	132.89	0.65	1.56E-06	1.21E-03
biological_process (GO:0008150)	19556	470	423.11	1.11	1.56E-06	1.32E-03
**developmental process (GO:0032502)**	4603	146	99.59	1.47	1.48E-06	1.38E-03
anatomical structure development (GO:0048856)	4410	140	95.41	1.47	2.36E-06	1.68E-03
cell-cell adhesion via plasma-membrane adhesion molecules (GO:0098742)	196	17	4.24	4.01	3.31E-06	2.19E-03
**nervous system development (GO:0007399)**	1707	67	36.93	1.81	4.68E-06	2.90E-03
neuron projection guidance (GO:0097485)	286	20	6.19	3.23	9.75E-06	5.32E-03
axon guidance (GO:0007411)	285	20	6.17	3.24	9.29E-06	5.39E-03
movement of cell or subcellular component (GO:0006928)	1116	48	24.15	1.99	1.42E-05	7.34E-03
cell development (GO:0048468)	1355	54	29.32	1.84	2.46E-05	1.20E-02
homophilic cell adhesion via plasma membrane adhesion molecules (GO:0007156)	166	14	3.59	3.9	3.19E-05	1.41E-02

locomotion (GO:0040011)	927	41	20.06	2.04	3.08E-05	1.43E-02
biological regulation (GO:0065007)	10578	278	228.86	1.21	3.43E-05	1.45E-02
neuron development (GO:0048666)	723	34	15.64	2.17	4.86E-05	1.96E-02
localization (GO:0051179)	3960	121	85.68	1.41	8.94E-05	3.46E-02
synaptic signaling (GO:0099536)	367	21	7.94	2.64	9.43E-05	3.50E-02
anatomical structure morphogenesis (GO:0009653)	2087	72	45.15	1.59	1.20E-04	4.14E-02
animal organ development (GO:0048513)	2579	85	55.8	1.52	1.19E-04	4.25E-02
**Reduced density group 2**						
**developmental process (GO:0032502)**	4603	215	131.12	1.64	9.57E-14	8.88E-10
anatomical structure development (GO:0048856)	4410	202	125.62	1.61	4.61E-12	2.14E-08
multicellular organism development (GO:0007275)	4073	189	116.02	1.63	1.17E-11	3.61E-08
multicellular organismal process (GO:0032501)	4925	217	140.29	1.55	2.44E-11	5.67E-08
anatomical structure morphogenesis (GO:0009653)	2087	115	59.45	1.93	3.93E-11	7.30E-08
cell differentiation (GO:0030154)	2335	123	66.51	1.85	9.88E-11	1.53E-07
cellular developmental process (GO:0048869)	2354	123	67.05	1.83	1.77E-10	2.35E-07
**cellular process (GO:0009987)**	14851	506	423.03	1.2	5.14E-10	5.31E-07
system development (GO:0048731)	3586	166	102.15	1.63	4.79E-10	5.56E-07
cell development (GO:0048468)	1355	81	38.6	2.1	1.32E-09	1.22E-06
**nervous system development (GO:0007399)**	1707	94	48.62	1.93	2.68E-09	2.26E-06
neurogenesis (GO:0022008)	1105	68	31.48	2.16	9.40E-09	7.27E-06
neuron development (GO:0048666)	723	51	20.59	2.48	1.84E-08	1.32E-05

cell morphogenesis (GO:0000902)	596	44	16.98	2.59	5.00E-08	3.31E-05
inorganic cation transmembrane transport (GO:0098662)	461	37	13.13	2.82	6.68E-08	3.87E-05
inorganic ion transmembrane transport (GO:0098660)	523	40	14.9	2.69	6.58E-08	4.07E-05
movement of cell or subcellular component (GO:0006928)	1116	65	31.79	2.04	1.51E-07	7.81E-05
locomotion (GO:0040011)	927	57	26.41	2.16	1.65E-07	8.06E-05
cation transmembrane transport (GO:0098655)	498	38	14.19	2.68	1.48E-07	8.09E-05
cellular component morphogenesis (GO:0032989)	567	41	16.15	2.54	2.15E-07	1.00E-04
generation of neurons (GO:0048699)	986	59	28.09	2.1	2.34E-07	1.04E-04
cell part morphogenesis (GO:0032990)	471	36	13.42	2.68	3.04E-07	1.28E-04
neuron differentiation (GO:0030182)	900	55	25.64	2.15	3.32E-07	1.34E-04
neuron projection development (GO:0031175)	558	40	15.89	2.52	3.62E-07	1.40E-04
plasma membrane bounded cell projection morphogenesis (GO:0120039)	460	35	13.1	2.67	4.89E-07	1.68E-04
cell morphogenesis involved in differentiation (GO:0000904)	514	38	14.64	2.6	4.67E-07	1.73E-04
neuron projection morphogenesis (GO:0048812)	460	35	13.1	2.67	4.89E-07	1.75E-04
cell projection morphogenesis (GO:0048858)	464	35	13.22	2.65	5.90E-07	1.96E-04
axon development (GO:0061564)	424	33	12.08	2.73	6.49E-07	2.01E-04
axonogenesis (GO:0007409)	403	32	11.48	2.79	6.40E-07	2.05E-04
**biological adhesion (GO:0022610)**	651	43	18.54	2.32	1.51E-06	4.37E-04

**cell adhesion (GO:0007155)**	651	43	18.54	2.32	1.51E-06	4.52E-04
ion transmembrane transport (GO:0034220)	752	46	21.42	2.15	3.03E-06	8.28E-04
cellular component organization (GO:0016043)	3851	158	109.69	1.44	3.01E-06	8.46E-04
regulation of biological quality (GO:0065008)	2034	95	57.94	1.64	3.48E-06	9.22E-04
cell morphogenesis involved in neuron differentiation (GO:0048667)	436	32	12.42	2.58	4.79E-06	1.24E-03
cellular component organization or biogenesis (GO:0071840)	4026	162	114.68	1.41	5.87E-06	1.43E-03
potassium ion transmembrane transport (GO:0071805)	171	18	4.87	3.7	5.79E-06	1.45E-03
axon guidance (GO:0007411)	285	24	8.12	2.96	6.38E-06	1.52E-03
metal ion transport (GO:0030001)	228	21	6.49	3.23	6.83E-06	1.55E-03
neuron projection guidance (GO:0097485)	286	24	8.15	2.95	6.74E-06	1.56E-03
plasma membrane bounded cell projection organization (GO:0120036)	878	50	25.01	2	8.75E-06	1.93E-03
inorganic cation import across plasma membrane (GO:0098659)	95	13	2.71	4.8	9.32E-06	1.97E-03
inorganic ion import across plasma membrane (GO:0099587)	95	13	2.71	4.8	9.32E-06	2.01E-03
cell communication (GO:0007154)	4175	166	118.92	1.4	1.00E-05	2.06E-03
cation transport (GO:0006812)	668	41	19.03	2.15	1.17E-05	2.36E-03
ameboidal-type cell migration (GO:0001667)	240	21	6.84	3.07	1.40E-05	2.77E-03
cell-cell adhesion (GO:0098609)	302	24	8.6	2.79	1.56E-05	3.02E-03

potassium ion transport (GO:0006813)	187	18	5.33	3.38	1.77E-05	3.36E-03
signaling (GO:0023052)	4133	163	117.73	1.38	1.85E-05	3.44E-03
cell projection organization (GO:0030030)	909	50	25.89	1.93	2.03E-05	3.69E-03
localization (GO:0051179)	3960	157	112.8	1.39	2.11E-05	3.77E-03
import across plasma membrane (GO:0098739)	104	13	2.96	4.39	2.23E-05	3.91E-03
transmembrane transport (GO:0055085)	1069	56	30.45	1.84	2.44E-05	4.19E-03
regulation of trans-synaptic signaling (GO:0099177)	175	17	4.98	3.41	2.71E-05	4.48E-03
modulation of chemical synaptic transmission (GO:0050804)	175	17	4.98	3.41	2.71E-05	4.57E-03
cell surface receptor signaling pathway (GO:0007166)	1312	65	37.37	1.74	3.04E-05	4.95E-03
calcium ion transport (GO:0006816)	162	16	4.61	3.47	3.85E-05	6.16E-03
lymph vessel morphogenesis (GO:0036303)	41	8	1.17	6.85	5.51E-05	8.66E-03
regulation of transcription, DNA-templated (GO:0006355)	2874	49	81.87	0.6	5.92E-05	8.86E-03
regulation of RNA biosynthetic process (GO:2001141)	2877	49	81.95	0.6	5.92E-05	9.00E-03
regulation of nucleic acid-templated transcription (GO:1903506)	2875	49	81.89	0.6	5.92E-05	9.15E-03
positive regulation of Wnt signaling pathway (GO:0030177)	70	10	1.99	5.02	7.16E-05	1.04E-02
calcium ion transmembrane transport (GO:0070588)	135	14	3.85	3.64	7.16E-05	1.06E-02
potassium ion homeostasis	32	7	0.91	7.68	8.63E-05	1.23E-02

(GO:0055075)						
regulation of RNA metabolic process (GO:0051252)	3055	54	87.02	0.62	9.28E-05	1.31E-02
inorganic ion homeostasis (GO:0098771)	451	29	12.85	2.26	1.02E-04	1.42E-02
lymph vessel development (GO:0001945)	59	9	1.68	5.36	1.04E-04	1.43E-02
positive regulation of catalytic activity (GO:0043085)	479	30	13.64	2.2	1.07E-04	1.44E-02
metal ion homeostasis (GO:0055065)	388	26	11.05	2.35	1.12E-04	1.48E-02
cellular homeostasis (GO:0019725)	457	29	13.02	2.23	1.20E-04	1.56E-02
import into cell (GO:0098657)	143	14	4.07	3.44	1.26E-04	1.58E-02
synaptic signaling (GO:0099536)	367	25	10.45	2.39	1.25E-04	1.59E-02
regulation of GTPase activity (GO:0043087)	220	18	6.27	2.87	1.25E-04	1.61E-02
cation homeostasis (GO:0055080)	441	28	12.56	2.23	1.59E-04	1.97E-02
potassium ion import across plasma membrane (GO:1990573)	63	9	1.79	5.02	1.64E-04	1.97E-02
positive regulation of molecular function (GO:0044093)	551	33	15.7	2.1	1.62E-04	1.98E-02
establishment of localization (GO:0051234)	3087	123	87.93	1.4	1.83E-04	2.15E-02
enzyme linked receptor protein signaling pathway (GO:0007167)	418	27	11.91	2.27	1.82E-04	2.17E-02
regulation of cellular biosynthetic process (GO:0031326)	3114	57	88.7	0.64	2.15E-04	2.50E-02
transmembrane receptor protein tyrosine kinase signaling pathway (GO:0007169)	275	20	7.83	2.55	2.36E-04	2.70E-02
signal transduction (GO:0007165)	3851	147	109.69	1.34	2.44E-04	2.76E-02
ion homeostasis (GO:0050801)	472	29	13.44	2.16	2.56E-04	2.87E-02

response to stimulus (GO:0050896)	5720	206	162.93	1.26	2.65E-04	2.93E-02
regulation of biosynthetic process (GO:0009889)	3134	58	89.27	0.65	2.81E-04	3.07E-02
cell-cell adhesion via plasma-membrane adhesion molecules (GO:0098742)	196	16	5.58	2.87	2.98E-04	3.14E-02
lymphangiogenesis (GO:0001946)	40	7	1.14	6.14	2.91E-04	3.14E-02
regulation of cell communication (GO:0010646)	1478	67	42.1	1.59	2.98E-04	3.18E-02
regulation of signaling (GO:0023051)	1484	67	42.27	1.58	3.11E-04	3.24E-02
regulation of macromolecule biosynthetic process (GO:0010556)	3082	57	87.79	0.65	3.17E-04	3.27E-02
regulation of cellular macromolecule biosynthetic process (GO:2000112)	3076	57	87.62	0.65	3.88E-04	3.95E-02
regulation of gene expression (GO:0010468)	3441	66	98.02	0.67	3.93E-04	3.96E-02
embryonic morphogenesis (GO:0048598)	713	38	20.31	1.87	4.01E-04	4.00E-02
response to mechanical stimulus (GO:0009612)	57	8	1.62	4.93	4.18E-04	4.13E-02
regulation of nucleobase-containing compound metabolic process (GO:0019219)	3132	59	89.21	0.66	4.41E-04	4.26E-02
cell volume homeostasis (GO:0006884)	19	5	0.54	9.24	4.41E-04	4.31E-02
regulation of catalytic activity (GO:0050790)	825	42	23.5	1.79	4.66E-04	4.46E-02
cell motility (GO:0048870)	690	37	19.65	1.88	4.90E-04	4.55E-02
cell migration (GO:0016477)	644	35	18.34	1.91	4.96E-04	4.56E-02
localization of cell (GO:0051674)	690	37	19.65	1.88	4.90E-04	4.60E-02
inositol lipid-mediated signaling	44	7	1.25	5.59	4.87E-04	4.62E-02

(GO:0048017)						
regulation of molecular function (GO:0065009)	1016	49	28.94	1.69	5.48E-04	4.98E-02

Table S5. Genomic variants represented in the populations. Total number and proportion of the different genomic variants represented in the complete genome of all individuals and the outliers of the fish reared under a baseline or reduced density.

	Complete genome		Baseline density outliers		Reduced density outliers	
Type of variant	Count	Percent	Count	Percent	Count	Percent
3' UTR variant	145947	0.54%	6	0.33%	5**	0.23%
5' UTR variant	53853	0.20%	2	0.11%	0**	0%
Downstream gene variant	2062370	7.66%	155	8.43%	186	8.47%
Intergenic region	1353405	5.03%	88	4.79%	110	5.01%
Intragenic variant	3269874	12.14%	267**	14.52%	278	12.66%
Intron variant	8575186	31.84%	580	31.54%	731	33.29%
Missense variant	263950	0.98%	10*	0.54%	5**	0.23%
Non codingtranscript exon variant	31997	0.12%	3	0.16%	0	0%
Non coding transcript variant	8944646	33.21%	590	32.08%	740	33.70%
Synonymous variant	154058	0.57%	7	0.38%	12	0.55%
Upstream gene variant	2000736	7.43%	131	7.12%	129**	5.87%

Asterisks represent significant difference compared to the complete genome (Fisher's Exact Test < 0.05). No difference was observed across density conditions.

Table S6. Families sequenced for the genomic analysis in each group.

Reduced density		Baseline density	
Captured	Escaped	Captured	Escaped
F10xM11	F10xM11	F10xM11	F11xM11
F11xM11	F11xM11	F10xM12	F11xM12
F11xM12	F12xM10	F11xM10	F12xM10
F12xM10	F12xM11	F11xM11	F12xM11
F12xM11	F12xM12	F11xM12	F1xM1
F12xM12	F1xM3	F12xM10	F1xM3
F1xM3	F2xM1	F12xM11	F2xM1
F2xM1	F2xM2	F12xM12	F3xM1
F2xM2	F3xM1	F1xM3	F3xM2
F3xM1	F3xM2	F2xM1	F3xM3
F3xM2	F4xM4	F2xM2	F4xM4
F4xM4	F5xM4	F3xM1	F4xM5
F4xM5	F5xM5	F4xM4	F5xM4
F5xM4	F5xM6	F4xM5	F5xM5
F5xM5	F5xM6	F5xM6	F5xM6
F6xM4	F6xM5	F6xM4	F6xM5
F6xM5	F7xM7	F6xM5	F7xM7
F7xM8	F7xM8	F7xM7	F7xM8
F7xM9	F7xM9	F7xM9	F7xM9
F8xM7	F8xM7	F8xM7	F8xM7
F8xM8	F8xM8	F8xM8	F8xM8
F8xM9	F8xM9	F8xM9	F8xM9
F9xM7	F9xM7	F9xM7	F9xM7
F9xM8	F9xM8	F9xM8	F9xM8

F represents the female identification number and M the male identification number.
